# A naturalistic study comparing the efficacy of unilateral and bilateral sequential theta burst stimulation in treating major depression – the U-B-D study protocol

**DOI:** 10.1186/s12888-023-05243-4

**Published:** 2023-10-10

**Authors:** Molly Watson, Arthur R. Chaves, Abir Gebara, Manon Desforges, Antoinette Broomfield, Noémie Landry, Alexandra Lemoyne, Stacey Shim, Jessica Drodge, Jennifer Cuda, Nasim Kiaee, Youssef Nasr, Christophe Carleton, Zafiris J. Daskalakis, Reggie Taylor, Lauri Tuominen, Ram Brender, Ruxandra Antochi, Lisa McMurray, Sara Tremblay

**Affiliations:** 1 University of Ottawa Institute of Mental Health Research at The Royal, 1145 Carling Ave, Ottawa, ON K1Z 7K4 Canada; 2 Department of Neuroscience, Carleton University, 1125 Colonel By Drive, Ottawa, ON K1S 5B6 Canada; 3 Faculty of Health Sciences, University of Ottawa, 125 University, Ottawa, ON K1N6N5 Canada; 4 School of Medicine, Stanford University, 300 Pasteur Drive, Stanford, CA 94305 USA; 5 Département de Psychoéducation Et Psychologie, Université du Québec en Outaouais, 283 Alexandre-Taché Boul, Gatineau, QC J8X 3X7 Canada; 6 Department of Psychiatry, University California San Diego, 9500 Gilman Dr, La Jolla, CA 92093 USA; 7 Department of Physics, Carleton University, 1125 Colonel By Drive, Ottawa, ON K1S 5B6 Canada; 8 Department of Psychiatry, University of Ottawa, 451 Smyth Road, Ottawa, ON K1H 8M5 Canada; 9 Royal Ottawa Mental Health Centre, 1145 Carling Ave, Ottawa, ON K1Z 7K4 Canada; 10 Department of Cellular and Molecular Medicine, University of Ottawa, 451 Smyth Road, Ottawa, ON K1H 8M5 Canada

**Keywords:** Major depressive disorder, Transcranial magnetic stimulation (TMS), Theta burst stimulation (TBS), Magnetic resonance spectroscopy, Evoked potential, TMS-EEG, Biomarkers, Predictors of response, Corticospinal excitability, Resting state connectivity

## Abstract

**Background:**

Major depressive disorder (MDD) is a prevalent mental health condition affecting millions worldwide, leading to disability and reduced quality of life. MDD poses a global health priority due to its early onset and association with other disabling conditions. Available treatments for MDD exhibit varying effectiveness, and a substantial portion of individuals remain resistant to treatment. Repetitive transcranial magnetic stimulation (rTMS), applied to the left and/or right dorsolateral prefrontal cortex (DLPFC), is an alternative treatment strategy for those experiencing treatment-resistant MDD. The objective of this study is to investigate whether this newer form of rTMS, namely theta burst stimulation (TBS), when performed unilaterally or bilaterally, is efficacious in treatment-resistant MDD.

**Methods:**

In this naturalistic, randomized double-blinded non-inferiority trial, participants with a major depressive episode will be randomized to receive either unilateral (i.e., continuous TBS [cTBS] to the right and sham TBS to the left DLPFC) or bilateral sequential TBS (i.e., cTBS to the right and intermittent TBS [iTBS] to the left DLPFC) delivered 5 days a week for 4–6 weeks. Responders will move onto a 6-month flexible maintenance phase where TBS treatment will be delivered at a decreasing frequency depending on degree of symptom mitigation. Several clinical assessments and neuroimaging and neurophysiological biomarkers will be collected to investigate treatment response and potential associated biomarkers. A non-inferiority analysis will investigate whether bilateral sequential TBS is non-inferior to unilateral TBS and regression analyses will investigate biomarkers of treatment response. We expect to recruit a maximal of 256 participants. This trial is approved by the Research Ethics Board of The Royal's Institute of Mental Health Research (REB# 2,019,071) and will follow the Declaration of Helsinki. Findings will be published in peer-reviewed journals.

**Discussion:**

Comprehensive assessment of symptoms and neurophysiological biomarkers will contribute to understanding the differential efficacy of the tested treatment protocols, identifying biomarkers for treatment response, and shedding light into underlying mechanisms of TBS. Our findings will inform future clinical trials and aid in personalizing treatment selection and scheduling for individuals with MDD.

**Trial registration:**

The trial is registered on https://clinicaltrials.gov/ct2/home (#NCT04142996).

**Supplementary Information:**

The online version contains supplementary material available at 10.1186/s12888-023-05243-4.

## Background

### Major depressive disorder

Major depressive disorder (MDD) stands among the leading causes of disability and poor quality of life, affecting ~ 264 million people worldwide [1, 2]. Among many, symptoms of MDD are characterized by persistent feelings of anxiety, sadness, hopelessness, and lack of motivation [3]. If left untreated these symptoms can be life-threatening; 31% of those affected by MDD will eventually attempt suicide [4]. MDD often has an early onset and becomes prevalent in young adults (~ 20–40 years-old) [5–7], adversely affecting the quality of life of those who are in the prime of their lives. MDD is a risk factor for other physical and psychological outcomes and is present in several other disabling conditions [8–10]. For these reasons, the socio-economic burden attributable to MDD is considered a global health priority [8, 10]. Despite the numerous and continuous growth of pharmacological and clinical therapies, treatment of MDD is yet empirical and challenging and their effectiveness is highly variable among individuals [11, 12]. In fact, approximately 35–50% of people with MDD are treatment resistant and do not respond to current standard antidepressant treatments [13].

### Transcranial magnetic stimulation for the treatment of MDD

In the early 90’s, transcranial magnetic stimulation (TMS) was created with the intent of using brief (~ 1 ms) single pulse magnetic fields, delivered through an insulated coil placed over the subject’s scalp, to painlessly and non-invasively activate cortical and spinal neurons and study human neurophysiology [14]. Rapidly after its creation, TMS became popular among research laboratories investigating central nervous system (CNS) function in healthy and clinical populations. In 1987, Brickford et al., first reported possible mood improvements in healthy subjects after being assessed with TMS for mechanistic investigation of corticospinal tract excitability [15]. This unexpected and intriguing finding paved the way for further research investigating the potential use of TMS to treat depression and treatment-resistant MDD. However, the antidepressant effects of magnetic pulses remained controversial until further technological advances of TMS stimulators. New stimulators capable of delivering high-frequency pulse rate (i.e., higher number of pulses per shorter period of time, Hz) were created, namely repetitive TMS (rTMS) [16, 17]. Different from TMS for mechanistic CNS assessment, rTMS was able to produce electrophysiological and behavioural changes associated with brain plasticity-like mechanisms [18]. In 1996, Pascual-Leone et al. reported the first convincing finding demonstrating mitigation of depressive symptoms on subjects with treatment resistant MDD following daily rTMS treatment [19].

**Fig. 1:**
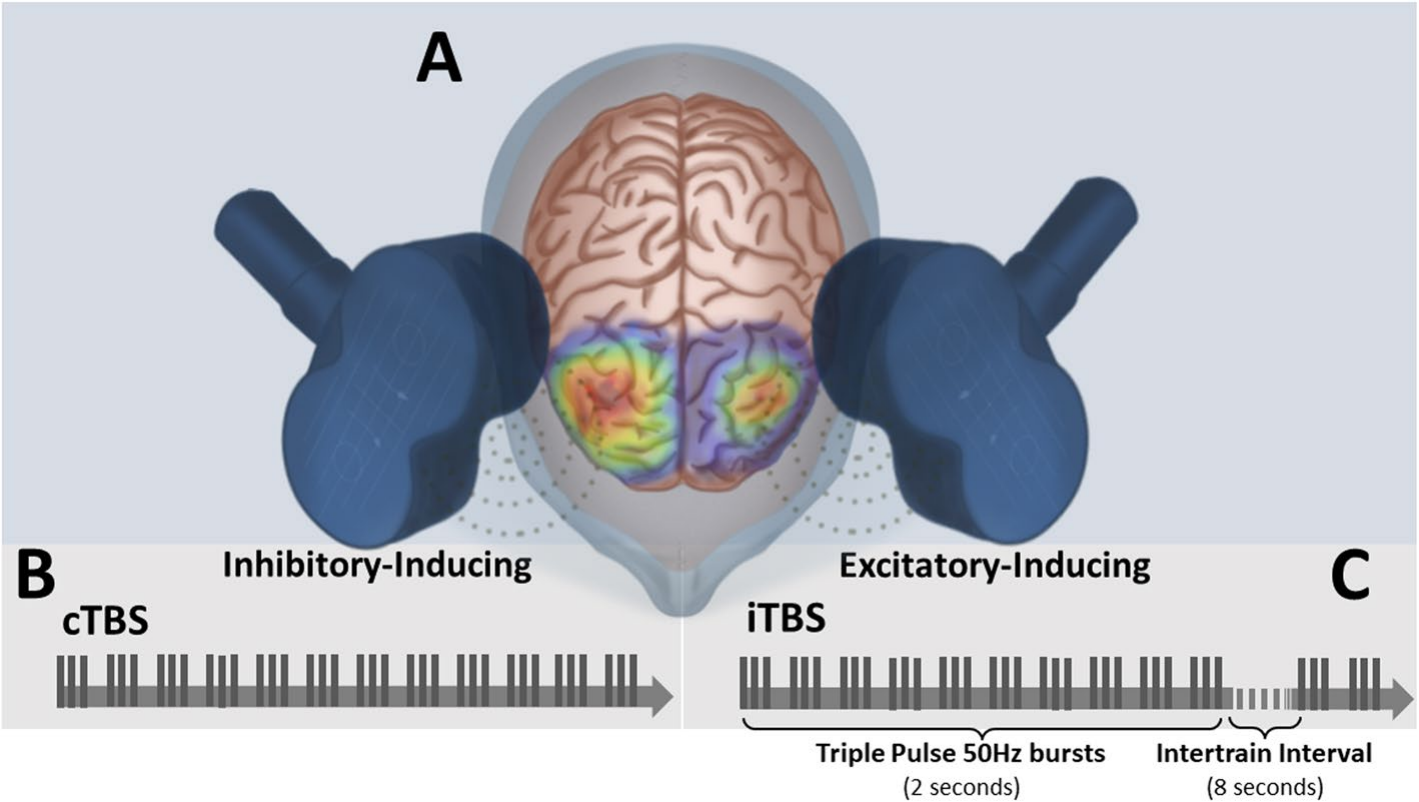
Representative figure demonstrating the (**A**) traditionally proposed asymmetry of dorsolateral prefrontal cortex (DLPFC) activity in people with a diagnosis of major depressive disorder (MDD; represented by the heatmap); lower activity in the left compared to the right DLPFC and degree of asymmetry have been associated with greater degree of depressive symptoms. **B** The inhibitory-inducing (i.e., long-term depression-inducing), continuous theta burst stimulation (cTBS), is employed to reduce the hyperactivity in the right DLPFC, whereas (**C**) the excitatory-inducing (i.e., long-term potentiation-inducing), intermittent theta burst stimulation (iTBS), is employed to increase activity in the hypoactivated left DLPFC. Original figure created by the co-author Dr. Arthur R. Chaves (Autodesk® Sketchbook® free software)

Performing rTMS to improve mood initially emerged from a conceptualization of an asymmetry of prefrontal cortex activity in individuals with a diagnosis of MDD [20]. Specifically, hypoactivation of the left and hyperactivation of the right dorsolateral prefrontal cortex (DLPFC) was believed to play a role in the pathophysiology of depression (Fig. 1A) [20]. Current and more advanced neuroimaging and brain connectivity studies have challenged this concept, and the neurobiological underpinnings of rTMS treatment have since evolved into a more complex network effect [21–28]. Grounded by these concepts, current rTMS protocols use high-frequency rTMS (HF-rTMS, e.g., 5-20 Hz) to increase left DLPFC activity, i.e., to promote long-term potentiation (LTP), and/or low-frequency rTMS (LF-rTMS, e.g., < 1 Hz) to decrease right DLPFC activity, i.e., to promote long-term depression (LTD) [29, 30]. In 2008, HF-rTMS over the left DLPFC was approved by the Food and Drug Administration (FDA) for individuals with treatment resistant MDD. This FDA-approved rTMS protocol, however, still showed some variable response rates [31] as not all participants responded to treatment. This points to the need for testing other stimulation strategies. Subsequent studies have been investigating the efficacy of other unilateral and bilateral rTMS protocols on superiorly treating MDD. Detailed current rTMS protocols and their results in MDD are well discussed in previous meta-analyses and reviews [32, 33]. Interestingly, the effectiveness of bilateral sequential stimulation, specifically HF-rTMS followed by LF-rTMS over the left and right DLPFC, did not surpass that of unilateral rTMS in reducing depressive symptoms [33]. Likewise, a recent large naturalistic study showed no difference between left unilateral and bilateral sequential rTMS on MDD [34]. Despite these results, the concept of unilateral vs bilateral sequential stimulation treatment provides a foundation for testing new and more refined ground-breaking brain stimulation techniques.

### Theta Burst Stimulation (TBS) – a newer rTMS protocol

In the early 2000’s a new pattern of rTMS was developed, namely TBS (Fig. 1B and C) in which the stimulus frequency and interstimulus interval would correspond to the electroencephalogram-assessed hippocampal theta waves that are linked with learning, memory formation, and LTP in humans [35]. Similar to LF-rTMS, continuous TBS (cTBS) decreases cortical activity through LTD-like plasticity [36] (Fig. 1B), whereas, similar to HF-rTMS, the intermittent pattern of TBS (intermittent TBS, iTBS) increases cortical activity through LTP-like plasticity (Fig. 1C).

A multi-site randomized non-inferiority trial involving over 400 individuals with a diagnosis of MDD compared iTBS over the left DLPFC vs HF-rTMS over the right side and showed similar efficacy between the two techniques on clinically decreasing depressive symptoms [37]. Building upon these findings, the same group showed, in a subsequent trial, that bilateral sequential TBS is non-inferior to standard bilateral sequential rTMS in individuals with MDD [38]. A major advantage of TBS is that its treatment session lasts only ~ 3–4 min to induce the comparable neuroplastic and antidepressant effects of a ~ 35–45 min rTMS session [39, 40]. Another difference between the two techniques is that iTBS typically utilizes lower (i.e., subthreshold) stimulation intensities, whereas rTMS uses higher (i.e., suprathreshold) stimulation intensities that can often bring some level of discomfort to participants [41]. Therefore, TBS might prove superior to rTMS as it is grounded on a more complex neurophysiological background, can be administered in a fraction of the time, it mitigates patients’ discomfort, and improves tolerability thus improving adherence to treatment. Those are especially important factors to consider as they might help encourage the use of non-invasive brain stimulation techniques in clinical settings.

### Biomarkers indexing the efficacy and response to rTMS treatment

Investigation of symptomology using standardized clinical assessments is essential to determine treatment efficacy and are usually the primary outcomes in clinical trials [37, 42, 43]. Additionally, the investigation of objectively measured biomarkers allows the elucidation of CNS’ underlying mechanisms (cellular/molecular) through which the therapy-induced rehabilitation is taking place and helps to identify and predict treatment response [44]. Thus, assessing predictive biomarkers is crucial in clinical trials. Neurophysiological investigation of non-invasive brain stimulation protocols traditionally relies on signal analysis of induced-motor evoked potentials (MEP) [45, 46]. For instance, MEP changes following rTMS performed over the primary motor area provide indexes of neurophysiology and brain plasticity [45–47]. However, motor outputs (e.g., MEP) might not index the induced-neurophysiological changes in non-motor regions such as in the rTMS-targeted DLPFC. Concurrent TMS and electroencephalography (TMS-EEG) to investigate TMS-evoked potentials (TEP) is a promising method to investigate plasticity changes outside motor regions that are more involved in depressive symptoms [48]. In individuals with a diagnosis of MDD, previous TMS-EEG research has shown that six weeks of HF-rTMS in the left DLPFC was shown to decrease the previously excessive gamma-aminobutyric acid (GABA) -mediated inhibition, a biomarker of hampered neuroplasticity [49, 50]. The anterior cingulate cortex (ACC) is another brain area that is believed to be involved in symptoms of depression. Previous resting state functional magnetic resonance imaging (fMRI) studies found that the antidepressant effects of rTMS treatment were predicted by the degree of connectivity between the stimulated site (i.e., DLPFC) and the ACC [51–54]. Levels of GABA and glutamate, assessed with magnetic resonance spectroscopy (MRS), have also been shown to be related to response to rTMS treatment [55, 56]. To date, no study has combined the measure of resting state fMRI, motor cortical inhibition/excitation assessed with TMS, prefrontal cortical inhibition/ excitation TMS-EEG, and GABA/glutamate levels in the ACC using MRS.

### Maintenance treatment

The clinical benefits of rTMS have been shown to last between three months [57] and one year [58], and there is limited knowledge on whether a continued maintenance rTMS therapy could prolong treatment efficacy. Promising results from one randomized study using rTMS responders demonstrated that relapse rates could be cut in half with an extra five months of rTMS maintenance therapy [59]. In addition, previous electroconvulsive therapy research showed that a flexible and participant-tailored maintenance schedule, where patients receive treatment based on symptomology, was superior to a schedule where patients receive treatment regardless of their symptoms [60]. To date, no bilateral sequential rTMS or iTBS study has performed a flexible maintenance protocol.

### Study objectives

No large-scale study has compared left unilateral versus bilateral sequential TBS for treating symptoms of MDD. The primary aim of this naturalistic, randomized double-blinded non-inferiority trial is to investigate the efficacy of unilateral (i.e., iTBS to the left DLPFC) and bilateral sequential (i.e., cTBS to the right DLPFC combined with iTBS to the left DLPFC) on reducing clinical symptoms of MDD. Based on previous research, we hypothesize that both treatments will effectively decrease depressive symptoms to a comparable degree (i.e., non-inferior) [33, 34, 61]. The incorporation of covariates during analysis may reveal a superiority of bilateral sequential TBS over unilateral treatment in specific subgroups. These subgroups include older individuals (≥ 60 years-old; [62], and/or individuals presenting with comorbid anxiety, and post-traumatic stress disorder [33, 63]). As mentioned, the clinical efficacy of rTMS is time limited [57, 58]. Therefore, this study will also investigate the efficacy of a six-month flexible maintenance phase. Additionally, to better understand the TBS-induced neurophysiological changes and their associations with therapeutic responses, multiple objectively measured neurophysiological variables will be assessed in this study. See Table 1 for a brief description of outcome measures and detailed hypotheses of this study. Ultimately, this study aims to: 1) inform whether unilateral TBS performed over the DLPFC is non-inferior to bilateral sequential TBS on treating MDD, 2) provide insight on neurophysiological mechanisms associated with treatment-induced effects and their longitudinal changes resulting from treatment, thus providing biomarkers of treatment response, and 3) explore neurophysiological markers, demographics, and clinical characteristics that may help identify responders and non-responders to treatment, paving the way for a more refined and personalized approach to TBS treatment.

**Table 1:** Outcomes and hypotheses of the study

Outcome	Rationale	Hypothesis
Clinical (primary)	Investigate changes in severity of depressive symptoms	Unilateral TBS will be non-inferior in comparison to bilateral sequential TBS on reducing depressive symptoms
Neurophysiological (secondary)	Investigate neurophysiological changes	Unilateral and bilateral sequential TBS will reduce the amplitude of the TMS-EEG measures (e.g., N100 and N45 amplitudes) in the left DLPFC, which were previously found to be enhanced in MDD [64]
Neurophysiological (secondary)	Explore predictors of outcome	The magnitude of changes in TMS-EEG measures after session 1 will strongly predict response to treatment Baseline levels of GABA/glutamate in the ACC will be associated with treatment response Baseline levels of resting state connectivity between the left DLPFC and ACC will be associated with treatment response
Covariates (exploratory)	Investigate response to treatment and include as controlling variables	Based on a recent large study using TBS in MDD [65–67], higher severity of depression will be associated with poorer clinical outcome, while low severity of depression and older age will be associated with a better clinical outcome. Bilateral sequential TBS will superiorly treat older individuals (≥ 60 years-old), individuals with PTSD and comorbid anxiety. Other covariates will be investigated (e.g., biological sex)

*ACC* Anterior cingulate cortex, *DLPFC* Dorsolateral prefrontal cortex, *MDD* Major depressive disorder, *PTSD* Post-traumatic stress disorder, *TBS* Theta burst stimulation, *TMS-EEG* Interleaved transcranial magnetic stimulation and electroencephalography

## Methods

### Study design and overview

**Fig. 2:**
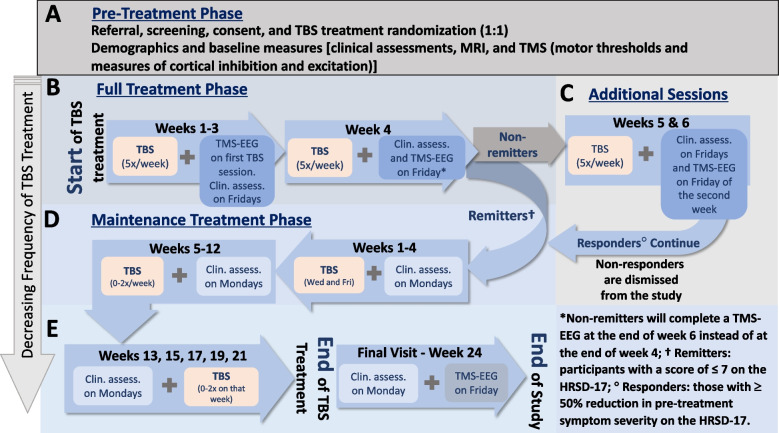
Study design and timeline of assessments and treatment schedule. **A** Pre-treatment phase: Following referral, screening, and consent, participants will be randomized into groups to receive theta burst stimulation (TBS) either bilateral sequentially or unilaterally. Participants will be assessed for demographics, complete baseline clinical measures (see Table 3), undergo magnetic resonance imaging (MRI), and transcranial magnetic stimulation (TMS) for motor threshold assessment and measures of cortical inhibition and excitation. **B** Full Treatment Phase: During weeks 1 to 3, participants will receive TBS treatment 5 × per week and clinical assessments will be performed every 5^th^ session. TMS paired with encephalography (TMS-EEG) will be performed during the first TBS treatment session. During week 4 clinical assessments will be performed at the second last treatment session, to determine if the participant reached remission. If so, TMS-EEG will be performed on the last treatment session and remitters will move to the next treatment phase, whereas (**C**) non-remitters will undergo two additional weeks of TBS treatment. Clinical assessments will be collected every 5^th^ session of the additional weeks, and a TMS-EEG will be performed at the last treatment session. Non-responders will be discharged from the study, whereas responders will move to (**D**) Maintenance Treatment Phase: During maintenance weeks 5–8, participants will be clinically assessed every Monday, and will undergo TBS treatment twice a week. During weeks 9–16, participants will receive 0, 1, or 2 sessions of TBS that week determined by their clinical assessment that week. (E) From week 17–25, participants will be clinically assessed every second week to determine whether they will receive 0, 1, or 2 TBS sessions that week. On week 28 a last clinical assessment and a last TMS-EEG session will be performed

This is a double-blinded randomized, naturalistic, non-inferiority trial comparing unilateral vs bilateral sequential TBS (iTBS in the left vs cTBS in the right followed by iTBS in the left DLPFC, respectively). Participants enrolling in this study are referred by their physician to receive rTMS for the treatment of MDD and are randomized in a 1:1 ratio to receive either unilateral or bilateral sequential TBS. The study design and timeline of assessments and treatment schedule is presented in detail in Fig. 2.

Following referral and screening (see Table 2 for detailed inclusion/exclusion criteria), written consent to participate will be obtained by trained research personnel after the participant has had the study explained to them in full and had the opportunity to ask questions. Participants will then be assessed for demographics and several clinical and neurophysiological outcomes (Fig. 2A). All demographic, clinical, and neurophysiological assessments throughout the study will be administered by trained research personnel. The clinical and neurophysiological outcome measures collected throughout the study (their description, assessment timepoint, and rationale for inclusion) are described in detail in Tables 3 and 4, respectively.

**Table 2:** Study inclusion and exclusion criteria

Inclusion Criteria	• Voluntary and competent to consent to the study • Able to communicate/read in English or French • Primary and/or predominant diagnosis of MDE without psychotic features confirmed by the Mini-International Neuropsychiatric Interview • Depressive symptoms not improving after ≥ 1 adequate doses of antidepressant trials in the current depressive episode • Moderate symptoms in the current depressive episode as indexed by a score of ≥ 15 on the HRSD-17 • Have been referred to rTMS treatment by their treating physician, and took a free and informed decision to follow this treatment • Are able to adhere to the treatment schedule • Have received a stable medication (including prescribed cannabis) or psychotherapy regiment for at least four weeks prior to entering the study • Have an education-adjusted score of ≥ 24 on the Mini-Mental State Evaluation if ≥ 65 years-old • Negative urine test for use of recreational drugs and/or pregnancy (tested at baseline)
Exclusion Criteria	• Current or past (< 3 months) substance (excluding caffeine or nicotine) or alcohol abuse/dependence, as defined in DSM-5 criteria. Based on the DSM-5 criteria, mild cannabis or alcohol use would be permissible in the past 3 months, moderate to severe would be an exclusion • Current use of illegal substances or cannabis (unless medical use)^a^ • Have a concomitant major unstable medical or neurologic illness (e.g., uncontrolled diabetes or renal dysfunction) • Organic cause to the depressive symptoms (e.g. thyroid dysfunctions), determined by the referring physician • Acute suicidality or threat to life from self-neglect • Are pregnant or breastfeeding, or thinking of becoming pregnant during course of treatment (pregnancy will be assessed by a urine test) • Have a specific contraindication for TMS (e.g., personal history of epilepsy or seizure, metallic head implant, pacemaker) • Unwilling to maintain current antidepressant regiment for the four weeks prior to and for the duration of the study • Are taking more than 1 mg of lorazepam or equivalent^b^ • Any other condition that in the opinion of the investigators, would adversely affect the participant’s ability to complete the study • Have failed a course of ECT within the current depressive episode (due to the lower likelihood of response to rTMS after ECT). If they have had failed ECT in the past, this does not exclude them

^a^Participants that have a prescribed dose of less than 3 g of cannabis or equivalent can remain on prescribed dosage for the duration of the study. However, we will ask participants not to use prescribed cannabis in the morning before each session, and to use a stable dosage for the duration of the study

^b^Patients that are prescribed a small dose of benzodiazepines (e.g. < 1 mg of lorazepam) will be allowed to participate, but patients and their treating physicians will be strongly encouraged to try reducing benzodiazepine dosage to the minimum possible dose in order to optimize performance prior to entering the study

*ECT* Electroconvulsive therapy, *MDE* Major depressive episode, *TMS* Transcranial magnetic stimulation, *rTMS* Repetitive TMS

**Table 3:** Clinical neuropsychological assessments

Assessment	Description/Purpose	Outcome Value	Timepoint of Collection
**MINI**^g^	Confirm diagnosis of MDE [68]	Yes/No	Baseline
**ATHF**^g^	History of antidepressant use and resistance [69]	Type, dosage	Baseline
**CONMED**^g^	Current medication use [70]	Yes (type, dosage)/No	Baseline
**HRSD-17**^e,g^	Severity of depression [71]	Score range: 0–53 (0–7 no depression; 8–13, mild depression; 14–18 moderate depression; 19–22, severe depression; ≥ 23 very severe depression)	Baseline, end of week 2 and 4 (and final week of additional treatment for non-remitters^d^); beginning of maintenance weeks 1–13, 15, 17, 19, 21, and final visit (week 24)
**MADRS**^f,g^	Severity of depression [72]	Score range: 0–60 (0–6, absence of symptoms; 7–19, mild depression; 20–34, moderate depression; 35–60, severe depression)	Baseline, end of week 4 (and final week of additional treatment for non-remitters^d^); final visit (week 24)
**SRRS**^g^	Depression-related psychomotor impairments [73]	Score range: 0–60	Baseline, end of week 4 (and final week of additional treatment for non-remitters^d^); final visit (week 24)
**YMRS**^f,g^	Presence and severity of manic or hypomanic symptoms [74, 75]	Score range: 0–60 (2, euthymia; 3, depression; 12, mania)	Baseline, end of week 2 and 4 (and final week of additional treatment for non-remitters^d^); beginning of maintenance weeks 1–13, 15, 17, 19, 21, and final visit (week 24)
**C-SSRS**^a,g^	Quantify suicidal ideation and behaviour [76]	5 items rated yes or no, with additional clarifying questions	Baseline and end of weeks 1–4 (and both weeks of additional treatment for non-remitters^d^); beginning of maintenance weeks 1–13, 15, 17, 19, 21, and final visit (week 24)
**MMSE**^b,^^g^	Presence of dementia [77]	Score range: 0–30 (30–26, could be normal; 25–20, mild; 19–10, moderate; 9–0, severe)	Screening visit
**Physician consultation**^g^	Verify general suitability for TBS; clinical oversight	N/A	Before participants consent to the study, end of week 4 (or final week of additional treatment for non-remitters^d^), end of maintenance, and/or as needed throughout the study
**QIDS-SR**_**16**_^f^	Depressive symptoms [78]	Score range: 0–27 (0–5, no depression; 6–10, mild depression; 11–15 moderate depression; 16–20 severe depression; ≥ 21, very severe depression)	Baseline, end of weeks 1–4 (and both weeks of additional treatment for non-remitters^d^); beginning of maintenance weeks 1–13, 15, 17, 19, 21, and final visit (week 24)
**ASRM**^f^	Manic or hypomanic symptoms [79]	Score range: 0–20 (0–5, low probability of mania; ≥ 6, high probability of manic or hypomanic condition)	Baseline, end of weeks 1–4 (and both weeks of additional treatment for non-remitters^d^)
**BAI**^f^	Severity of anxiety [80]	Score range: 0–63 (0–21, low anxiety; 22–35, moderate anxiety; ≥ 36 potentially concerning levels of anxiety)	Baseline, end of weeks 1–4 (and both weeks of additional treatment for non-remitters^d^); beginning of maintenance weeks 1–13, 15, 17, 19, 21, and final visit (week 24)
**BSS**	Suicidal ideations and behaviors [81]	Score range: 0–38 (higher scores indicate higher suicide risk)	Baseline, end of weeks 1–4 (and both weeks of additional treatment for non-remitters^d^); beginning of maintenance weeks 1–13, 15, 17, 19, 21, and final visit (week 24)
**Q-LES-Q-SF**^f^	Enjoyment and satisfaction in routine activities [82]	Score range: 14–70. Raw scores are transformed into a percentage maximum possible score	Baseline, at the end of week 4 (and final week of additional treatment for non-remitters^d^); final visit (week 24)
**WEMWBS**^f^	Overview of mental well-being [83]	Score range: 7–35 (higher scores indicate higher positive mental wellbeing)	Baseline, at the end of week 4 (and final week of additional treatment for non-remitters^d^); final visit (week 24)
**PSQI**	Quality and pattern of sleep prior to the start of treatment [84]	Score range: 0–21 (0, no difficulty; 21 severe difficulty in all areas)	Baseline, at the end of week 4 (and final week of additional treatment for non-remitters^d^); final visit (week 24)
**LSEQ**	Sleep quality [85]	Score range: 0–100 (visual analog scale)	Baseline, end of weeks 1–4 (and both weeks of additional treatment for non-remitters^d^); beginning of maintenance weeks 1–13, 15, 17, 19, 21, and final visit (week 24)
**PCL-5**^fc^	Post-traumatic stress disorder symptoms [86]	Score range: 0–80 (scored pre- post treatment; 5–10 point change, reliable change not due to chance; 10–20 point change, clinically significant change)	Baseline, end of weeks 1–4 (and both weeks of additional treatment for non-remitters^d^); beginning of maintenance weeks 1–13, 15, 17, 19, 21, and final visit (week 24)
**TSSS**	To assess rate of sleepiness in the moment [87]	Score range: 1–7	Baseline, end of week 4 (and final week of additional treatment for non-remitters^d^); final visit (week 24)
**IPAQ-SF**	To assess levels of physical activity and sedentarism [88]	Continuous data: Duration (e.g., minutes) and frequency (e.g., days) of activities performed and multiple of resting metabolic rates (e.g., energy expenditure). Categorical data: 1) inactive, 2) minimally active, and 3) highly active	Baseline, end of week 4 (and final week of additional treatment for non-remitters^d^); final visit (week 24)

*ASRM* Altman Self-Rating Mania Scale, *ATHF* Antidepressant Treatment History Form, *BAI* Beck Anxiety Inventory, *BSS* Beck Scale for Suicidal Ideation, *CONMED* Concomitant Medication Log, *C-SSRS* Columbia-Suicide Severity Rating Scale, *HRSD-17* Hamilton Rating Scale for Depression 17-item, *IPAQ-SF* International Physical Activity Questionnaire—Short Form, *LSEQ* Leeds Sleep Evaluation Questionnaire, *MADRS* Montgomery–Asberg Depression Rating Scale, *MINI* Mini International Neuropsychiatric Interview, *MMSE* Mini Mental State Evaluation, *PCL-5* Posttraumatic Stress Disorder Checklist for DSM-5, *PSQI* Pittsburgh Sleep Quality Index, *QIDS-SR16* 16-item Quick Inventory of Depressive Symptoms –self report, *Q-LES-Q-SF* Quality of Life Enjoyment and Satisfaction Questionnaire – Short Form, *SRRS* Salpêtrière Retardation Rating Scale, *TSSS* The Stanford Sleepiness Scale, *WEMWBS* Short Warwick Edinburgh Mental Well-Being Scale, *YMRS* Young Mania Rating Scale

^a^After baseline assessment, only administered if BSS shows active suicidal ideation

^b^Administered only for participants who are > 65 years-old

^c^Administered only if comorbid post-traumatic stress disorder diagnosis

^d^Non-remitters: participants who do not demonstrate a score of ≤ 7 (i.e., no depression) in the 17-item Hamilton Depression Rating Scale (HRSD-17)

^e^Primary outcome measure

^f^Secondary outcome measure

^g^Outcome measures that are administered by trained personnel

**Table 4:** TMS and neuroimaging techniques used in the study

Technique	Experiment/Protocol	Purpose/proposed neurophysiology	Targeted CNS Structure	Primary Reason for Collecting
**rTMS (TBS)**	iTBS	Promotes LTP-like neuromodulation (i.e., excitatory-inducing)	Left DLPFC	Treatment Intervention
	cTBS	Promotes LTD-like neuromodulation (i.e., inhibitory-inducing)	Right DLPFC	Treatment Intervention
**Single and Paired-Pulse TMS**	RMT	Assesses cortical excitability via indirect activation of cortical interneurons (I-wave)	M1 (bilaterally)	Used to normalize the following TMS experiments (e.g., % of RMT)
	AMT	Cortical excitability via direct activation of corticospinal tract neurons (D-wave)	M1 (bilaterally)	Used to normalize the TBS treatment intensity (e.g., % of AMT)
	MEP Amplitude	Capacity of the corticospinal tract in recruiting neurons from faster temporospatial summation at cortico-motoneuronal synapses. Reflects glutamatergic (NMDA- and AMPA-receptor) activity	Left M1	Used as unconditioned MEP (i.e., test MEP), to calculate degree of inhibition during paired-pulse experiments (e.g., degree of inhibition = conditioned/unconditioned MEP)
	SICI	Short-interval intracortical inhibition primarily influenced by GABA_A_-receptor activity	Left M1	To investigate TMS biomarkers of treatment response examined with correlation analysis between baseline TMS and pre-post changes in clinical measures
	LICI	Long-interval intracortical inhibition primarily influenced by GABA_B_-receptor activity	Left M1	
	ICF	Intracortical facilitation primarily influenced by glutamatergic (NMDA- and AMPA-receptor) activity	Left M1	
**TMS-EEG**	TEP and ERSP	Assesses trans-synaptic activation of local and distal cortical networks mediated by excitatory and inhibitory cortical circuitry	TMS performed on the left and right DLPFC (120% of RMT) and TEP/ERSP are recorded from the DLPFC (primarily) and whole brain (exploratorily)	To investigate TMS-EEG biomarkers of treatment response, examined with correlation analysis between baseline TEPs/ERSPs and pre-post changes in clinical measures. To investigate cortical changes from treatment, examined with repeated-measures analysis [pre- vs post initial treatment (week 4, or week 6 for non-remitters), vs post maintenance phase (e.g., final week (week 24)]
**MRI**	Structural MRI	Assesses anatomy and morphology of CNS structures	Whole brain	To be uploaded into the neuronavigation software to assist with the location and targeting of the M1 and DLPFC during TMS assessments and TBS treatment, respectively (MRI-assisted TMS)
	MRS	Availability of neurotransmitters within the CNS (GABA and Glutamate)	Bilateral ACC	To investigate biomarkers of treatment response, examined with correlation analysis between baseline values and pre-post changes in clinical measures
	Functional MRI	Resting state connectivity between CNS structures	Bilateral DLPFC and ACC	

*ACC* Anterior cingulate cortex, *AMT* Active motor threshold, *cTBS* Continuous TBS, *CNS* Central nervous system, *DLPFC* Dorsolateral prefrontal cortex, *EEG* Electroencephalogram, *ERSP* Event related spectral perturbation, *ICF* Intracortical facilitation, *iTBS* Continuous TBS, *LICI* Long-interval intracortical inhibition, *LTD* Long-term depression, *LTP* Long-term potentiation, *M1* Motor cortex, *MEP* Motor evoked potential, *MRI* Magnetic resonance imaging, *MRS* Magnetic resonance spectroscopy, *RMT* Resting motor threshold, *rTMS* repetitive transcranial magnetic stimulation, *SICI* Short-interval intracortical inhibition, *TBS* Theta burst stimulation, *TES* TMS-evoked potential, *TMS* Transcranial magnetic stimulation

The TBS treatment will be administered by trained research personnel 5 days a week (Monday-Friday) over the first 4 weeks (initial treatment phase; Fig. 2B). Participants who do not achieve remission (i.e., non-remitters) after the initial treatment phase as per the 17-item Hamilton Rating Scale for Depression (HRSD-17; score of > 7) will undergo two additional weeks of treatment (totaling 6 weeks of initial treatment) (Fig. 2C). The additional two weeks provides an opportunity for individuals, particularly older adults, who require a longer period to achieve response from stimulation treatments [89]. Participants who do not respond to treatment (i.e., < 50% decrease in pre-treatment HRSD-17 score) after the two additional weeks of full treatment will be discharged from the study and treatment recommendations will be provided to their referring physician by the study psychiatrist. Participants who show remission at week 4 or response at week 6 will enter a six-month maintenance phase, where treatment will be determined based on their symptomatology (Fig. 2D and E). In the first 4 weeks of this phase, participants will be remotely assessed for clinical outcomes (phone or video-call) once per week (e.g., on Mondays) and treatment will be administered 2x/week (e.g., every Wednesday and Friday). In the following 8 weeks (i.e., weeks 9–16) treatment frequency will be further decreased, and the HRSD-17 score, remotely assessed weekly on Mondays, will determine the number of treatment sessions that week. Participants can receive 0, 1, or 2 treatment sessions, as follows: a) no TBS session, if HRSD-17 score of ≤ 8 (no depression) or a < 3 point increase from their final initial treatment HRSD-17 score; b) 1 TBS session, if their HRSD-17 score increased by ≥ 3 and < 8 from their final initial treatment HRSD-17 score; c) 2 TBS sessions, if their HRSD-17 score increased by ≥ 8 from their final initial treatment HRSD-17 score [60]. From week 17–25, participants will be clinically assessed on Mondays of every second week to determine whether they will receive 0, 1, or 2 TBS sessions that week based on the same criteria previously mentioned. Finally, in week 28 participants will complete clinical assessments and TMS-EEG on Monday and Friday, respectively, they will not receive TBS treatment.

Throughout the study participants will be reminded of the importance of following study guidelines and adhering to the treatment schedule. Study staff will do their best to accommodate for participants' schedules and mitigate conflicts keeping them from adhering to the study protocol. Missed treatment sessions (e.g., due to illness or scheduling conflicts) will be made up for at the end of the treatment phase to ensure the proposed total number of treatment sessions are completed. Participants will be discharged from the study if they are non-adherent, i.e., fail to attend or are absent for > 3 consecutive treatment days or 15% of sessions during the treatment phase. Missed treatments relating to Covid-19 related symptoms will not be counted towards this criterion. Participants will also be discharged if they have a sustained relapse in the maintenance phase of the study, i.e., requiring two treatments per week for more than two consecutive assessments.

### Ethics and registration

All protocols and patient informed consent for this study have been approved by the Research Ethics Board (REB) of The Royal's Institute of Mental Health Research (REB# 2,019,071 approved on August 7, 2019). Significant changes to this study (e.g., any procedure, inclusion/exclusion criteria) will only take place after Ethics Committee’s approval. The trial is registered on https://clinicaltrials.gov/ct2/home (#NCT04142996). All in-person study procedures will take place at the University of Ottawa Institute of Mental Health Research at The Royal.

### Participants

A maximum of 256 participants (128 per group; biological male or female, ≥ 18 years-old) with a diagnosis of a major depressive episode (MDE) will be recruited through advertisements posted at the Royal Ottawa Mental Health Centre (ROMHC) and medical clinics in the Ottawa/Gatineau region. All participants must have a referral from their treating physician (e.g., psychiatrist or general physician). Multiple strategies will be used to ensure participant retention. Specifically, communication and engagement will be maintained through regular contact, education and feedback channels. Participants will be compensated for their involvement in the study. Monitoring will be diligent, with frequent assessments and adherence tracking. In cases of discontinuation or protocol deviations, exit interviews, data collection extensions and adverse event reporting will ensure that data is obtained.

### Randomization and blinding

Participants are randomized (1:1 ratio) using the study randomizer software (https//www.studyrandomizer.com). A permutated block is used to randomize participants into the treatment groups. Participant stratification is based on the following variables: 1) age (< 65 or ≥ 65 years-old), 2) depression (unipolar or bipolar), 3) comorbid generalized anxiety (yes or no), 4) comorbid post-traumatic stress disorder (yes or no), and 5) biological sex (male or female). Upon entering these participant characteristics, the software provides a number (e.g., 0 or 1) that corresponds to a treatment group in which experimenters and participants are blinded to. Participants will remain assigned to their designated group until the treatment concludes.

This study is a two-arm double-blind trial as neither the participants nor the outcome assessor, care provider and data analysts will know the condition assigned to the participants. The master randomization list of the treatment blinding will be kept by a scientist of the research centre that is not involved in the project. This can be broken in case of emergency (e.g., severe adverse events such as seizure induced by treatment) and they can then inform required medical professionals directly if necessary. Directly involved research staff will be blinded (including the principal investigator and study psychiatrists).

### Unilateral and bilateral treatment – iTBS and cTBS

The TBS parameters used in this study are consistent with previous determined guidelines [90]. Specifically, TBS will consist of triple-pulse 50-Hz bursts applied at a rate of 5 Hz using a MagPro X100-MagOption stimulator device (Magventure, Farum, Denmark). iTBS will be delivered through a B70 coil and cTBS will be delivered using an active/sham B65 figure-of-eight cooled-coil (Magventure, Farum, Denmark). Unilateral TBS consists of 40 s of sham cTBS applied to the right DLPFC followed by 190 s of iTBS (i.e., excitatory-inducing) over the left DLPFC. Bilateral sequential TBS will consist of 40 s of cTBS (i.e., inhibitory inducing) over the right DLPFC followed by 190 s of iTBS over the left DLPFC. Stimulation intensity for both iTBS and cTBS will be 80% of the individual's active motor threshold (AMT) assessed with single-pulse TMS (for AMT protocol, see *Single- and Paired-Pulse TMS* section below). Magnetic resonance imaging (MRI) -assisted neuronavigation will ensure proper coil position and targeting of the participants’ DLPFC. The TMS coil will be positioned with the handle pointing backwards and at a 45-degree angle from the midline and centered against the left and right DLPFC locations (e.g., Fig. 1). The DLPFC stereotactic coordinates (MNI-152) are: x = -38, y = 44, z = 26 [37].

To ensure the sham stimulation mimics the somatosensory components experienced during active TBS stimulation (e.g., pulse noise, tingling, tapping sensation), the sham coil will produce the same auditory feedback and will deliver synchronous electrical pulses to the scalp through electrodes. To ensure double-blinding, participant IDs will be entered in a software-controlled switch that will automatically select the previously randomized TBS condition (active or sham) created by a team member not involved in treatment and data collection. To evaluate the integrity of the blinding procedure, at the end of each treatment phase (i.e., initial and maintenance phase), participants will complete a questionnaire to assess their opinion on whether they received active stimulation on one or both sides of their head. A questionnaire assessing 12 common acute side-effects of rTMS (e.g., headache, pain, fatigue) will be given after each TBS session to monitor potential side effects. These questions are: 1) in the 24 h following your most recent TMS session, did you experience back or neck pain? 2) pain or discomfort at the stimulation site? 3) headache? 4) tinnitus? 5) dizziness? 6) fatigue (more than usual)? 7) insomnia? 8) anxiety or agitation? 9) nausea? 10) vomiting? 11) migraine aura? 12) abnormal sensations?". Participants rate symptoms on a scale ("none," "very mild," "mild," "moderate," or "severe") and indicate if they attribute this rating to the treatment (“yes”, “no"). A post-treatment feedback questionnaire will be administered at the end of the first and last TBS session in the initial treatment phase to assess the quality of the study and include questions such as 1) "did you feel welcome when coming into the lab?", 2) "was everything thoroughly explained to you prior to treatment?", 3) "did you feel that you knew what to expect coming into the study?", 4) "how can we improve to make the experience better?". Participants rate these questions as “not at all," “small degree," “neutral," “moderate degree," or “high degree." If there are any adverse events recorded during these questionnaires or during any study visit, we will record them and report them to the REB.

### Study outcomes

#### Clinical outcomes

Clinical outcomes will be determined using self-report questionnaires and clinical interviews conducted by trained research personnel. Several standardized questionnaires were selected to provide a comprehensive understanding of depressive-related symptoms and will serve as outcomes of treatment response. See Table 3 for all clinical outcomes collected, their description/purpose, outcome value, and timepoint of collection.

##### Primary outcome measure

Response to treatment will be primarily defined as a ≥ 50% reduction in pre-treatment symptom severity as measured by the HRSD-17 score. Treatment remission will be defined as a HRSD-17 score ≤ 7. These definitions of treatment response and remission are based on recent literature assessing the effects of rTMS in individuals with depression [33, 37, 38, 91, 92].

##### Secondary outcome measure

For secondary analyses response to treatment will be defined as a ≥ 50% reduction in pre-treatment symptom severity as measured by the mean 16-item Quick Inventory of Depressive Symptoms–self report (QIDS-SR_16_) score and Montgomery–Asberg Depression Rating Scale (MADRS). Remission will be defined as a QIDS-SR_16_ score of ≤ 6 and a MADRS score < 12. Additionally, other secondary outcomes will include effect of treatment on anxiety (Beck Anxiety Inventory), symptoms of mania (Young Mania Rating Scale, Altman Self-Rating Mania Scale), quality of life (Quality of Life Enjoyment and Satisfaction Questionnaire), symptoms of post-traumatic stress disorder (Post-traumatic Stress Disorder Checklist for DSM-5), and well-being (Short Warwick Edinburgh Mental Well-Being Scale).

#### MRI – Brain connectivity and metabolism

Within two weeks prior to treatment onset, whole brain MRI from participants will be collected to assist with the targeting of the DLPFC, TMS-EEG, and TBS sessions. MRI scans will be performed using a 3 T Siemens mMR Integrated Whole-Body PET/MR Scanner and 32-channel head coil (Ceresensa Inc.). First, a T1-weighted (MEMPRAGE, 1mm^3^ isotropic voxel-size, lasting approximately 6 min) scan will be collected, followed by two runs of BOLD-EPI (TR = 2300 ms, 27 mm^3^ isotropic voxel-size, 183 volumes/run, 8 min per run) to collect resting state images. Lastly, a single voxel MRS scan will be collected using the GABA-edited MEGA-PRESS sequence [93] with the following parameters: TR = 2000 ms, TE = 68 ms, acquisition bandwidth = 2000 Hz, pulse placement edit-on/edit-off = 1.9/7.5 ppm, number of excitations edit-on/edit-off = 64/64 for a total of 128 averages. The voxel will be positioned over the bilateral ACC (35 × 20x20mm) using the T1 images and according to previous literature [94, 95]. Two MEGA-PRESS acquisitions edited with GABA will be acquired (4.36 min each) as well as two water-unsuppressed reference scans (0.48 min each).

Standard resting state fMRI data preprocessing and denoising steps will be conduted using fMRIPrep (https://fmriprep.org/) and the functional connectivity toolbox (CONN: www.nitrc.org/projects/conn). Preprocessing steps will include correction for motion, functional realignment and unwarping, direct normalization into the MNI common space, and smoothing with a spatial filter. Seed region-of-interest will be defined as a 10 mm radius circle around the TMS target regions. Correlations between the residuals in the seed and the whole brain time series will be calculated for each fMRI run separately. Runs will be combined by calculating a voxel/vertex–wise mean of z-transformed r-values. Resting state fMRI connectivity values between the seed and the ACC will be extracted with a region of-interest in the ACC (dorsal, pregenual, subgenual). For MRS data, post-processing of MRS signals will be conducted using Matlab scripts (MathWorks, USA) and LCModel [96]. The LCModel software will be used to quantify the concentrations of GABA and Glutamate + Glutamine (Glx) in the ACC. Individual resonances having Cramer-Rao Lower Bounds greater than 20% will be omitted from further investigation. Moreover, exclusion of data will be made on spectra that exhibit obvious artifacts, such as large head motions, lipid contamination (huge peak near 1 ppm) and poorly shimmed data. Post-processing will include phase correction and frequency drift correction of the individual sub-spectra utilizing residual water as a reference, followed by the average of the phase- and frequency-corrected spectra. GABA and Glx levels will be obtained from difference and “EDIT OFF” spectra, respectively.

#### Single- and paired-pulse TMS – corticospinal excitability

Participants’ MRI will be uploaded into the neuronavigation software (Brainsight, Rogue Research Inc, Montreal, QC) to assist with visualization of participants’ primary motor cortex and guide coil position during the TMS and TBS sessions (i.e., neuronavigated MRI-assisted TMS). Single-pulse TMS will be performed using a MagPro X100-MagOption Stimulator with a 70 mm figure-of-eight coil (MagVenture, Denmark) to elicit MEPs in the contralateral first dorsal interosseous (FDI) muscle. Biphasic pulses will be used to determine treatment active thresholds, and monophasic pulses will be used to determine resting thresholds for TMS biomarkers. MEPs will be collected using the neuronavigation built-in electromyography (EMG) system (Brainsight, Rogue Research). Surface electrodes (Kendall™, Cardinal Health, Waukegan, USA) placed over the belly of the FDI muscles will collect MEPs of both hands. The reference and ground electrodes will be placed in the interphalangeal joint of each index fingers and on any prominent bone (e.g., olecranon, medial/lateral epicondyle, styloid process), respectively, ipsilaterally to the tested FDI muscle.

Using the uploaded MRI in the neuronavigation software, a grid with targets (9 × 9, 3 mm distance between targets) was a priori centered on the central sulcus. Each target received three-five suprathreshold stimulations, and the "hotspot" for the contralateral FDI was determined based on the target that yielded the highest average peak-to-peak MEP amplitude. Next, participants' resting and active motor thresholds (RMT and AMT, respectively) will be determined using the relative frequency estimation method (for detailed protocol see; [97]. In brief, the least amount of stimulation intensity to elicit five out of ten 50 µV amplitude MEPs during complete relaxation and 200 µV amplitude MEPs while participants perform 10% of their maximal pinch contraction determines RMT and AMT, respectively. RMT and AMT will be used to normalize the following experiments across participants and to individualize the stimulation intensity for the TBS treatment, respectively (e.g., % of RMT and AMT).

TMS biomarkers indexing corticospinal inhibition and excitation, such as MEP amplitudes, short- and long-interval intracortical inhibition (SICI, LICI, respectively), and intracortical facilitation (ICF), will be collected during the first TMS session after motor threshold are determined. Baseline MEP amplitudes will be collected with single-pulse TMS at a stimulation intensity of 130% of RMT. SICI, LICI, and ICF will be assessed with paired-pulse TMS, where the conditioned stimulus precedes a second stimulus that is separated by an interstimulus interval (ISI). For SICI and ICF, the conditioned stimulus, delivered at 80% of RMT, is followed by a test stimulus, delivered at 130% of RMT, at an ISI of 2 and 12 ms, respectively. For LICI, both the conditioned and test stimuli are delivered at 130% of the RMT, and the ISI is 100 ms. Degree of inhibition is measured as the ratio between the single-pulse TMS elicited- and the paired pulse-TMS elicited- MEPs (e.g., = conditioned/unconditioned MEP); lower ratios signify higher degree of intracortical inhibition [97]. A total of 60 MEPs will be collected; 20 single- and 60 paired-pulses (20 per paired-pulse measure).

#### TMS-EEG: DLPFC activity

To investigate the acute effects of TBS on the DLPFC activity, TEPs captured with electroencephalography (EEG; i.e., TMS-EEG), will be collected before and after the first and last TBS session of the initial treatment phase (i.e., session 1 and session 20 for remitters, or session 30 for individuals receiving an additional 2 weeks of treatment), and before and after the last treatment session of the maintenance phase. This will be done to investigate treatment-induced long-term changes of TBS induced-plasticity in both DLPFCs. A TMS-compatible 64-channel EEG cap (ActiCap Slim, Brainvision, Gmb) will be positioned on participant's head. Conductive gel will be inserted in each electrode on the EEG cap using a syringe. Impedance levels will be kept under 5 kΩ. An ActiCHamp Plus EEG system and Brain Vision Recorder software (BrainVision, Gmb) will record EEG signals at a sampling rate of 5000 Hz. Electrodes will be referenced online to CPz and grounded to FPz. To minimize TMS-induced artefacts, EEG wires will be arranged prior to recordings according to Sekiguchi et al. 2011 [98]. To minimize auditory and somatosensory potentials, a white noise which includes specific time-varying frequencies of the TMS click will be delivered through sound-reducing earbuds during EEG recordings and sound-reducing ear defenders will be positioned on top of the earbuds, as recommended by Rocchi et al. 2021 [99]. To reduce coil vibration to the skin, a thin layer of foam will be placed under the coil. Finally, a questionnaire assessing the level of sensory perception using a Likert scale (i.e., sound, vibration, muscle contraction) will be administered after each TMS-EEG session.

RMT and AMT will be re-investigated after the placement of the EEG cap due to the added distance to the scalp. Participants will be asked to close their eyes and a four-minute resting EEG will be recorded. Next, pre and immediately post the participant's regular TBS treatment, a block of 80 single-pulse TMS at a stimulation intensity of 120% of RMT will be delivered over the left and right DLPFC and TEPs will be recorded.

EEG data will be analyzed using custom scripts on Matlab platform (R2021, Mathworks, USA) and toolboxes including EEGLAB [100] and Fieldtrip [101]. The signal will be epoched around the TMS pulse and baseline corrected to TMS free data. Surrounding TMS pulse signal will be removed and interpolated. Noisy channels and epochs will be removed via an automated script [102]. Two rounds of independent component analysis (ICA) will be performed with EEGLAB *fastica* algorithm to remove the TMS-related artefacts (i.e., ICA1: high amplitude activation removal) and other sources of artifacts (i.e., ICA2: eye movements, eye blinks, muscle activation, and other TMS-related artifacts removal). Bandpass and notch filters will be applied. Removed channels will be interpolated and data will be re-referenced to the average scalp electrodes. TEPs will be measured at the location of stimulation and on a whole-brain basis and amplitudes of TEPs will be compared across time at the typical time windows (N15, P30, N45, P60, N100, and P200). TMS-evoked oscillatory power, namely event related spectral perturbation (ERSP), will be measured by converting TEPs into the frequency domains using Morlet wavelet decomposition, and power values will be averaged in the delta to gamma frequency bands at the typical time windows. TBS-induced DLPFC activity changes will be determined by the pre-post differences in amplitude of TEPs and power of TMS-evoked cortical oscillations over the left and right DLPFC. See Table 4 for a full list of TMS and neuroimaging techniques used in the study, their purpose/proposed neurophysiology, targeted CNS structure, and primary reason for collecting.

## Data analysis

All statistical tests will be two-tailed, and significance (*p*-value) will be set at α = 0.05. All analyses will be performed on SPSS (Chicago, US) and/or using the open-source R statistical software (R core Team, Vienna, Austria and the R-Studio package rstatix; Kassambara, 2022). All data will be reported descriptively to summarize population characteristics. Unless otherwise stated, continuous data will be reported as mean and standard deviation (mean ± SD) and dichotomous variables will be reported as frequency (i.e., percentage) values.

All participants will be included in the analyses ‘as-randomized’ (i.e., bilateral and unilateral TBS) and data will be analyzed regardless treatment adherence and drop-outs. To this end, missing values in the data set (due to participants missing appointments, drop-out/discharge), if present, will be appropriately managed during analysis as per previous suggested procedures for clinical research [103, 104].

### Data management

Except for the screening forms, the consent forms and the master code list, all data will be identified exclusively by an anonymous identification code. This code will be attributed following the phone screening, prior to the first visit. Following this, all documents will contain only de-identified/anonymous data. Paper copy of the Informed Consent Forms will be kept in a locked filing cabinet under the responsibility of the Principal Investigator (ST). The Research Electronic Data Capture (REDCap; https://www.project-redcap.org/) will be used for clinical data collection and overall study data management over the course of this project. Participant interview and self-report data will be entered directly in a case report form (CRF) using the REDCap [105] and secured by multiple levels of authentication. Administrative privileges will be given to a Data Manager who will be responsible for design of CRFs, data entry management and quality control. All data will be password-protected and saved in a secured server. These will include the EEG and TMS raw data, master randomization list, the subject identification code list and the subject enrollment list. Automatic backups of electronic data will be performed daily.

To assure double-blinding, the master randomization list will be created by a scientist of the research centre that is not involved in the research project. A paper copy will be kept in a sealed envelope stored in a locked filing cabinet and a password-protected electronic copy will be kept on a flash drive in a sealed envelope stored in a locked filing cabinet.

Qualtrics will only be used as a means to store participant answers to the MINI assessment. This will be conducted on a laptop that is kept in a locked cabinet. Qualtrics uses TLS or Transport Layer Security encryption (also known as HTTPS) for all their data that is transmitted through their platform. Our Qualtrics account is password protected. Qualtric services are hosted by data centres that are trusted as they are audited independently using the SSAE-18 method (an industry standard).

As per the Canada Tri-Council Policy Statement: Ethical Conduct for Research Involving Humans (TCPS-2), a data monitoring committee is not required for this trial. Auditing will be conducted yearly from the principal investigator. The Royal’s Research Ethics Board will also conduct auditing of trial conduct during the course of the trial.

In accordance with our commitment to transparency and the ethical conduct of this clinical trial, a comprehensive plan for disseminating trial results to various stakeholders was established. Firstly, we intend to communicate trial outcomes to participants, ensuring they receive understandable summaries of the study's findings in plain language. Healthcare professionals and researchers will be informed through open-access peer-reviewed publications in relevant medical journals and presentations at local, national, and international conferences. Findings will also be reported in publicly accessible clinicaltrial.gov database.

### Sample size

Based on our power calculations and the findings from exemplary studies, our sample size offers sufficient statistical power for the planned statistical tests. For example, Plewnia et al. (2014), in *n* = 32 (16/group), found clinically meaningful differences in response to treatment rate (≥ 50% reduction in baseline MADRS score) when comparing 6 weeks of bilateral sequential vs sham TBS (56% vs 25%, respectively) [42]. In *n* = 60 (15/group), Li et al. (2014) showed that 2 weeks of bilateral sequential TBS had superior effectiveness in treating depressive symptoms (≥ 50% reduction in baseline HRSD-17 score) when compared to unilateral (iTBS or cTBS) or sham TBS [106]. For our primary (non-inferiority) statistical analysis (i.e., analysis of covariance, ANCOVA), a total sample size of *n* = 158 was calculated [Gpower Software V.3.1 [107]; power 0.80, α = 0.05, expected effect size *Cohen’s f* = 0.25 (medium; Cohen, 1988)]. For our analysis of predictors of response (i.e., regression analysis), a total sample size of *n* = 30–39 will be required for a regression model that includes 3–6 independent (and/or controlling) variables predicting 30% (R^2^) of variance in the outcome (dependent) variable (Gpower Software V.3.1 [107]; power 0.80, α = 0.05, expected effect size *Cohen’s f*^*2*^ = 0.43) [108]. All other statistical tests are exploratory and, therefore, no sample size calculations were performed. Therefore, in order to achieve the calculated sample size (*n* = 158), we aim to recruit a minimum of 171 individuals, considering an anticipated dropout rate of 6–8% based on previous research [37]. Our recruitment efforts will be capped at a maximum of 256 participants, aligning with our capacity to provide TBS treatment over a five-year timeframe.

### Non-inferiority analysis – unilateral vs bilateral sequential TBS

As per Blumberger, et al. (2018), a non-inferiority analysis will be performed to investigate whether the proposed bilateral sequential TBS is non-inferior to the current FDA-approved unilateral iTBS [37]. An ANCOVA will be performed with the baseline HRSD-17 as controlling (i.e., covariate) and the final HRSD-17 score as the dependent outcome variable. The null hypothesis is that the baseline-adjusted final HRSD-17 scores for unilateral and bilateral stimulation will be comparable (i.e., non-inferior) [33, 34, 61]. Although, the incorporation of covariates (i.e., older individuals and individuals presenting with comorbid anxiety and/or post-traumatic stress disorder) during analysis may reveal a superiority of bilateral sequential TBS over unilateral treatment. During ANCOVA, potential missing data will be addressed using multiple imputation methods [104].

### Effectiveness of TBS treatment

Secondarily, the effectiveness of both unilateral and bilateral TBS as well as their difference on treating MDD will be investigated with generalized linear mixed models with treatment groups (unilateral vs bilateral TBS) as between-subjects factor, and outcome measures (clinical scores, neurophysiological measures) as within-subject factors. The main effects of Time (pre, post) and Time X Group (unilateral vs bilateral TBS) interaction will be investigated, and Bonferroni-adjusted pairwise comparisons will be performed to determine statistically significant within- and between-subjects differences. This analysis take into account missing data, avoiding the use of additional methods such as multiple imputations. The binominal outcomes will also be examined using Chi-Squared tests, and the difference in proportion rates between the groups will be reported (e.g., Groups: bilateral vs unilateral TBS, and outcome: % of response/remission rate identified as “yes” and “no” as per HRSD-17 ≤ 50% reduction from the baseline score [37, 43, 106]. If the sample size and their demographics allow for group stratification based on age groups, biological sex and gender, and the presence/absence of comorbidities, we will conduct additional between-subject analyses to compare groups.

### Predictors of treatment response

Exploratory relationships across outcome measures will be investigated using Pearson’s or Spearman’s correlation coefficients for normal and non-normal distributed data, respectively. Specifically, relationships between baseline levels of neurotransmitters, cortical excitability, and capacity of plasticity (rsfMRI and MRS, TMS, and TMS-EEG, respectively) and the degree of change (∆ pre-post treatment) in the assessed clinical outcomes will be performed to identify potential neurobiological predictors of response to treatment. As well, to provide some insight on possible neurobiological mechanisms involved in response to treatment, correlation analyses will be conducted between the degree of change (∆ pre-post treatment) in cortical plasticity (TMS-EEG) and clinical treatment response (e.g., HRSD-17). Additionally, regression analyses will be performed to predict response to treatment (e.g., “responders” and “non-responders”, and HRSD-17) with the neurobiological outcomes (MRI, TMS, TMS-EEG) entered as predicting variables. We will include demographics (e.g., age, sex, levels of education, amount of benzodiazepines being taken, employment status (measure of functionality), baseline HRSD-17 (MDD symptom severity), baseline BAI anxiety, degree of resistance to pharmacotherapy, etc.) as controlling variables. To investigate predictors within each treatment group, the correlations and regression analyses will be performed separately for each group and/or with groups entered as predicting variables. A Receiver Operating Characteristic (ROC) curve analysis will be performed to investigate the ability of significant predictors to classify response to treatment (achieved remission, "yes" or "no", based on ≥ 50% reduction from baseline HRSD-17 score; dependent variable). The area under the ROC curve with 95% CI will be investigated and the ‘cut-off point’ best classifying remission and its respective sensitivity and specificity percentage values will be identified.

Biological sex will be considered during analyses. Biological sex (male, female) will be inputted as predicting outcome variables (dependent) during regression analysis predicting the independent variables. As well, differences in treatment response and neurophysiological measures between the two biological sexes will be explored using independent t-tests.

Interim analyses of TMS, TMS-EEG, MRI, and clinical data will be conducted to further explore biomarkers of depression and comorbid disorders and mechanisms of action of TBS. These will further enhance the impact of the study and leverage the large dataset collected. These analyses will be blinded to treatment allocation and trial outcome (e.g., without dividing response or remission rates by group) until the end of the trial. Objective and hypotheses will be guided by existing knowledge and new discoveries in the field of non-invasive brain stimulation and psychiatry.

## Discussion

The main objective of this work is to investigate the efficacy of unilateral vs bilateral sequential TBS on treating MDD, the number one leading cause of disability worldwide [1]. Promising findings have shown that unilaterally delivering either excitatory- or inhibitory-inducing non-invasive brain stimulation to the left and right DLPFC, respectively, can significantly reduce MDD symptoms [46]. Other findings have shown that performing bilateral sequential stimulation, i.e., exciting the left and inhibiting the right DLPFC in the same session, is a therapy that may offer superior therapeutic effects in specific subpopulations of individuals with MDD such as people over the age of 65 [33]. Compared to HF-rTMS, TBS may be a superior non-invasive brain stimulation technique for treating MDD as it is faster and uses lower stimulation intensities [37, 38]. This naturalistic randomized large-scale non-inferiority trial is the first study investigating unilateral vs bilateral sequential TBS in treating MDD. On top of a relatively longer period of treatment, one notable strength of our study is the addition of a maintenance-phase protocol where the frequency of treatment is customized according to the individual's clinical progress. In addition, because of the heterogeneity of MDD it is expected that there will be variability of treatment response across participants. By collecting multiple symptoms using clinically valid assessments and multiple neurophysiological biomarkers – and to our knowledge, one of the largest set ever collected to date in a single study – our findings will: 1) establish whether the tested protocols (unilateral vs bilateral sequential TBS) differ in efficacy in treating MDD, 2) identify biomarkers of treatment response that are crucial to assist with participant stratification in future clinical trials, and most importantly, during participant selection for TBS treatment in clinical settings, and 3) help to elucidate CNS mechanisms through which the therapy is taking place, thus providing insight for the development of better targeted non-invasive brain stimulation protocols.

### Unilateral vs bilateral rTMS/TBS for the treatment of treatment resistant MDD

Despite the existence of effective treatments for MDD, individuals classified as treatment resistant encounter difficulties in finding suitable treatment options. Both unilateral and bilateral rTMS have shown some efficacy in treating treatment resistant MDD [34, 37, 42, 106]. However, no large-scale naturalistic study has directly compared bilateral sequential and unilateral TBS to treat MDD. This is especially important to explore considering the research trend on investigating synergetic effects of combined brain stimulation therapies. However, to date, current studies fail to identify effective bilateral brain stimulation protocols for MDD. For example, recent studies report that the effectiveness of bilateral sequential stimulation, involving HF-rTMS followed by LF-rTMS over the right and left DLPFC, did not exceed that of unilateral rTMS in reducing depressive symptoms [33, 34]. Expanding on these existing knowledge gaps, the Blumberger group reported bilateral TBS compared to standard bilateral rTMS achieved non-inferior symptom reduction for depression in individuals with a diagnosis of MDD [38]. Our study on the antidepressant effects of unilateral and bilateral TBS serves as a valuable addition to these ongoing research efforts.

### Maintenance treatment protocol

We are assessing the efficacy of a 6-month flexible maintenance phase, as previous rTMS and TBS research has shown that relapse and symptom re-emergence can occur 6 months after cessation of a successful treatment [58, 59, 109]. One rTMS study has shown that including a maintenance phase could significantly lower relapses rate in MDD, in comparison to those who did not receive maintenance treatment [59]. Though, there are very few rTMS controlled and open-label studies that have been published on maintenance after successful response to a shorter-term treatment [110], and no studies on TBS maintenance. Also, as previously suggested, the frequency and schedule of treatment administration needs to be participant-tailored and delivered based on each individual symptom severity when transitioning from the acute to maintenance treatment phases [109, 111]. Therefore, one of the important goals of this study is to determine the efficacy of a flexible maintenance TBS treatment phase that is delivered based on each participant’s symptomology and clinical development, i.e., improvement or worsening of symptoms. Participants will be evaluated with several clinically valid tests throughout the study to gain a full comprehensive data set of depressive and linked symptoms (see Table 3). This will help us to identify distinct symptom changes from the two TBS treatments (i.e., unilateral vs. bilateral).

### Brain biomarkers – assessing CNS effects and predicting treatment response

In clinical research, efforts have been recently made to improve upon the practice of delivering treatments in a ‘*one size fits all*’ perspective [44]. This is the reason why exploring cross-modal biomarkers (e.g., clinical characteristics/neuroimaging/neurophysiology) is highly encouraged in clinical trials as they would help to identify participants who would and would not benefit from treatment, and provide insight on the underlying cause behind the different responses [44, 112, 113]. We expect that not every participant will respond clinically to the TBS treatments [114, 115], therefore investigating biomarkers is an important aspect of this study. The multiple neurophysiological and neuroimaging experiments collected will help us to identify predictors of response that could serve to guide participant selection for TBS treatment, and guide future non-invasive brain stimulation research on the development of novel protocols for participants profiled as non-responders.

Biomarkers indexing effectiveness of non-invasive brain stimulation protocols traditionally rely on investigation of corticospinal excitability assessed by TMS-evoked MEPs. Analysis of its EMG features provide indexes of excitatory and inhibitory receptor activity involved in LTP and LTD [116, 117], processes of strengthening and weakening synaptic connections, respectively, in which neuroplasticity relies [118]. Several studies have shown that, although probed in the motor cortex (M1), MEPs are associated with functionality and symptoms inside and outside motor regions, such as cognition, migraines, sleep deprivation, cognitive impairment, pain, and others [117, 119–124]. For this reason, assessment of MEPs is largely used in research attempting to understand CNS-induced changes resulting from various interventions and, especially, to provide biomarkers of disease, symptom progression, and recovery in the lesion-disrupted brain (e.g., stroke, Multiple Sclerosis, Alzheimer’s, Parkinson’s, mild cognitive impairment, and others [116, 117, 125–127]).

In our study, we are performing typical single- and paired-pulse TMS experiments to assess biomarkers of intracortical inhibition and excitation that are primarily, but not exclusively, mediated by GABAergic and glutamatergic neurotransmission, respectively [97]. Specifically, we will collect motor thresholds (RMT and AMT), biomarkers of cortical excitation (MEP amplitudes and ICF), and biomarkers SICI and LICI [97]. These biomarkers are of particular importance since previous TMS studies have shown that they are sensitive to abnormalities associated with MDD and related symptoms (e.g., cognitive impairments, mood alterations) [128]. For instance, studies investigating TMS motor thresholds in depressive disorders have shown reduced corticospinal excitability in the left cortex [129, 130] and interhemispheric imbalance, i.e., lower and higher excitability in the left and right cortex, respectively [131]. Delivering rTMS to excite the DLPFC has shown to revert this lowered excitability and reduce interhemispheric asymmetries, effects that were associated with clinical improvements [50]. As for the TMS biomarkers indexing GABAergic-mediated corticospinal inhibition (e.g., SICI, LICI) [97], previous studies have suggested a possible cortical disinhibition (i.e., decreased SICI) in the left hemisphere in MDD [132, 133]. These findings are interesting since outside pathology, excitation and inhibition are not mutually exclusive; lower excitation is typically followed by higher inhibition and vice-versa [64]. The fact that this does not seem to be the case in MDD points to the value of TMS-assessing both inhibitory and excitatory circuitry for a better comprehension of the treatment induced-neurophysiological effects and to identify complementary biomarkers of treatment response in MDD. The limitation of relying solely on TMS-induced MEPs to investigate the effects of interventions not focusing on the motor cortex (i.e., focusing on the DLPFC) is that this brain region is not amongst the most relevant CNS structures involved in symptoms of MDD and is not the targeted brain region of this treatment. Complementary to MEPs, we hope to improve our treatment predictability by including other neurophysiological assessments such as TMS-EEG and MRI. These techniques will help us to investigate the TBS-induced neurophysiological changes within the treatment-targeted region (i.e., DLPFC) and connected CNS structures closely associated with depressive symptoms (e.g., ACC).

For instance, a previous TMS-EEG study has shown higher intracortical inhibition (e.g., N45 and N100 amplitude) in the DLPFC of individuals with a diagnosis of MDD in comparison to healthy controls. N45 amplitudes were also predictive of a higher degree of depression [64]. By looking at other events of TEPs (e.g., P60, a biomarker of intracortical excitation) these authors also reported higher excitation and an excitation/inhibition imbalance in the DLPFC of individuals with a diagnosis of MDD in comparison to healthy controls, and a further abnormal lack of association between excitation and inhibition in MDD, an association that is present in the healthy brain [64]. Abnormalities in neurophysiological features in MDD have shown to be associated with diminished capacity for neuroplasticity, which in turn, has been associated with a higher degree of MDD symptoms and relapses [128]. Ge et. al (2022) showed that the first treatment session of rTMS-inhibitory stimulation delivered to the right DLPFC induced acute widespread changes in functional connectivity measured by fMRI (i.e., TMS-fMRI) [134]. Interestingly, these authors reported that this first acute rTMS-induced plasticity response, but not resting state fMRI, was predictive of clinical outcomes (determined by MADRS scores) collected 4 weeks later, after cessation of daily rTMS treatment [134]. Most recently, in a subset of MDD participants who completed the Blumberger trial, Strafella, et al. (2023) reported that both iTBS and HF-rTMS reduced N100. Interestingly, participants who responded to treatment had higher baseline N100 and higher N45 post treatment when compared to non-responders [135]. Taken together, these findings provide support for the link between the neurophysiological effects of TBS and treatment effectiveness. Therefore, we will use TMS-EEG to investigate trans-synaptic activation of local and distal cortical networks mediated by excitatory and inhibitory cortical circuitry [48]. One novelty of our TMS-EEG method is the additional investigation during treatment, which has never done before and could complement the existing pre-post findings and could identify new neurophysiological markers of TBS response. As well, like Ge et. al (2022) we will assess the widespread TBS treatment-induced plasticity throughout treatment (see Table 3 for time points) [134]. This will be done to investigate whether the first treatment-induced plasticity is predictive of outcomes and whether the longer-term treatment can restore the diminished plasticity in MDD. As well, we will include additional neuroimaging techniques assessed during baseline, MRS and resting state fMRI, to investigate the availability of neurotransmitters (GABA and glutamate) within the ACC and connectivity between CNS structures, respectively. Both of these techniques are popular in MDD research and have shown to be sensitive to identifying abnormal neurophysiological features in MDD (for review see [136, 137]).

### Limitations and strengths

#### Limitations

This study is not exempt from limitations. First, as with many clinical conditions, MDD manifests in various forms, and this heterogeneity will lead to differences in treatment response. Also, there is no consensus on optimal stimulation schedule of treatments when treating MDD. While daily treatment is commonly used in clinical practice and in most research studies, the evidence supporting its superiority over other treatment regiments is not definitive. Accelerated treatment approaches that involve delivering multiple TBS sessions in a condensed time frame can offer advantages, particularly for individuals in need of immediate relief or those with other barriers (e.g., jobs, living far away from the treatment site). An example of such an approach is the 'Stanford accelerated intelligent neuromodulation therapy', which consists of 10 daily TBS sessions [138] and has demonstrated the ability to achieve remission of MDD symptoms within five days. Therefore, a limitation of this study is that we did not explore the effects of different treatment regiments, and that one treatment per day may not be optimal for all participants. Further investigation encompassing a broader range of treatment schedules is needed.

#### Strengths

The strengths of this study are noteworthy. First, a major strength is the naturalistic nature of the study leading to a sample of participants that is representative of the treatment resistant population being treated in rTMS clinics (e.g., with comorbidities, bipolar depression, etc.). This will offer a highly rich dataset allowing for analyses of subsamples. Secondly, we incorporated multiple assessments using standardized clinical scales with existing cut-off criteria for diagnosis determination that will help us obtain a more comprehensive symptomatic profile from participants. Another strength of our study is the inclusion of a maintenance phase that individualizes treatment frequency based on ongoing clinical assessments. This approach recognizes the importance of tailoring the treatment schedule to the specific needs of each participant, promoting a personalized approach to therapy. Also, the utilization of neuronavigation and participants' own MRI scans for targeting the DLPFC offers significant benefits in guiding and personalizing treatment. MRI- and neuronavigated-assisted TBS enhances the precision and accuracy of targeting, potentially improving treatment outcomes for the participants in this study and contributes to the scientific rigor and specificity of our research. Finally, despite debates on stimulation intensities and their potential for enhanced neuromodulatory effects, evidence suggests that exceeding 70% of RMT (~ 85% of AMT) may not be optimal for effective TBS neuromodulation [139]. Therefore, our selection of the standard intensity at 80% of AMT may be optimal and avoid potential unnecessary side-effects related to higher stimulation intensities.

#### Clinical implications

This study aims to investigate the efficacy of unilateral versus bilateral sequential TBS in treating MDD. Previous research has shown promising results with unilateral stimulation of the left or right DLPFC, while bilateral sequential stimulation has shown potential benefits in specific subpopulations. This newer rTMS protocol (TBS), with its faster treatment time and lower stimulation intensities, offers advantages over its predecessor, rTMS. This naturalistic randomized trial is the first to compare unilateral and bilateral TBS in MDD treatment. The inclusion of a maintenance-phase protocol and comprehensive assessment of symptoms and neurophysiological biomarkers will strengthen this study’s findings. The results will contribute to understanding the differential efficacy of the tested protocols, identifying biomarkers for treatment response, and shedding light on the underlying mechanisms of TBS therapy. Our findings will inform future clinical trials and aid in personalized treatment selection for individuals with a diagnosis of MDD.

### Supplementary Information

**Additional file 1:** **Appendix I.** World Health Organization Trial Registration Data Set. **Appendix II.** SPIRIT guidelines: REB Revision Chronology. **Appendix III.** SPIRIT 2013 Checklist: Recommended items to address in a clinical trial protocol and related documents*. **Appendix IV.** Informed Consent Form for Participation in a Research Study.

## Data Availability

All Individual Participant Data collected from this study will be de-identified for all parties who have permission to access it. This de-identified data may be shared with other researchers at the Royal's Institute of Mental Health Research, as stated in the consent form. De-identified and analysis code may be shared with other researchers upon request addressed to the principal investigator.

## Abbreviations

ACC: Anterior cingulate cortex
ANCOVA: Analysis of covariance
AMT: Active motor threshold
ASRM: Altman Self-Rating Mania Scale
ATHF: Antidepressant Treatment History Form
BAI: Beck Anxiety Inventory
BSS: Beck Scale for Suicidal Ideation
CONMED: Concomitant Medication Log
CNS: Central nervous system
C-SSRS: Columbia-Suicide Severity Rating Scale
cTBS: Continuous theta burst stimulation
DSM-5: Diagnostic and Statistical Manual of Mental Disorders, Fifth Edition
DLPFC: Dorsolateral prefrontal cortex
ECT: Electroconvulsive therapy
EEG: Electroencephalography
EMG: Electromyography
ERSP: Event related spectral perturbation
FDA: Food and Drug Administration
FDI: First dorsal interosseous
fMRI: Functional magnetic resonance imaging
GABA: Gamma aminobutyric acid
Glx: Glutamate + Glutamine
HF-rTMS: High-frequency repetitive transcranial magnetic stimulation
HRSD-17: 17-item Hamilton Rating Scale for Depression
ICA: Independent component analysis
ICF: Intracortical facilitation
IPA-SF: International Physical Activity Questionnaire-Short Form
ISI: Interstimulus interval
iTBS: Intermittent theta burst stimulation
LF-rTMS: Low-frequency transcranial magnetic stimulation
LICI: Long-interval intracortical inhibition
LSEQ: Leeds Sleep Evaluation Questionnaire
LTD: Long-term depression
LTP: Long-term potentiation
MADRS: Montgomery–Asberg Depression Rating Scale
MEP: Motor evoked potentials
MDD: Major depressive disorder
MDE: Major depressive episode
MINI: Mini International Neuropsychiatric Interview
MMSE: Mini Mental State Evaluation
MRI: Magnetic resonance imaging
MRS: Magnetic resonance spectroscopy
M1: Motor cortex
PCL-5: Posttraumatic Stress Disorder Checklist for DSM-5
PSQI: Pittsburgh Sleep Quality Index
QIDS-SR-16: 16-item Quick Inventory of Depressive Symptoms-self report
Q-LES-Q-SF: Quality of Life Enjoyment and Satisfaction Questionnaire-Short Form
SSRS: Salpêtrière Retardation Rating Scale
REB: Research ethics board
RMT: Resting motor threshold
ROC: Receiver Operating Characteristic
ROMHC: Royal Ottawa Mental Health Centre
rTMS: Repetitive transcranial magnetic stimulation
SICI: Short-interval intracortical inhibition
TBS: Theta burst stimulation
TEP: Transcranial magnetic stimulation evoked potentials
TMS: Transcranial magnetic stimulation
TMS-EEG: Interleaved transcrainal magnetic stimulation and electroencephalography
TSS: The Stanford Sleepiness Scale
WEMWBS: Short Warwick Edinburgh Mental Well-Being Scale
YMRS: Young Mania Rating Scale
µV: Microvolts
kΩ: Kiloohm

## References

[1] Friedrich MJ (2017). Depression is the leading cause of disability around the world. JAMA.

[2] James SL, Abate D, Abate KH, Abay SM, Abbafati C, Abbasi N (2018). Global, regional, and national incidence, prevalence, and years lived with disability for 354 diseases and injuries for 195 countries and territories, 1990–2017: a systematic analysis for the Global Burden of Disease Study 2017. The Lancet.

[3] van Loo HM, Aggen SH, Kendler KS (2022). The structure of the symptoms of major depression: Factor analysis of a lifetime worst episode of depressive symptoms in a large general population sample. J Affect Disord.

[4] Dong M, Zeng LN, Lu L, Li XH, Ungvari GS, Ng CH (2019). Prevalence of suicide attempt in individuals with major depressive disorder: a meta-analysis of observational surveys. Psychol Med.

[5] Lee CT, Chiang YC, Huang JY, Tantoh DM, Nfor ON, Lee JF (2016). Incidence of Major Depressive Disorder: Variation by Age and Sex in Low-Income Individuals. Medicine (Baltimore).

[6] Mojtabai R, Olfson M, Han B (2016). National trends in the prevalence and treatment of depression in adolescents and young adults. Pediatrics.

[7] Patten SB, Wang JL, Williams JV, Currie S, Beck CA, Maxwell CJ (2006). Descriptive epidemiology of major depression in Canada. Can J Psychiatry.

[8] Ferrari AJ, Charlson FJ, Norman RE, Patten SB, Freedman G, Murray CJL (2013). Burden of depressive disorders by country, sex, age, and year: findings from the global burden of disease study 2010. PLoS Med.

[9] Greenberg PE, Fournier AA, Sisitsky T, Simes M, Berman R, Koenigsberg SH (2021). The economic burden of adults with major depressive disorder in the United States (2010 and 2018). Pharmacoeconomics.

[10] Gutiérrez-Rojas L, Porras-Segovia A, Dunne H, Andrade-González N, Cervilla JA (2020). Prevalence and correlates of major depressive disorder: a systematic review. Braz J Psychiatry.

[11] DeRubeis RJ, Siegle GJ, Hollon SD (2008). Cognitive therapy vs. medications for depression: Treatment outcomes and neural mechanisms. Nat Rev Neurosci.

[12] Schatzberg AF, Rush AJ, Arnow BA, Banks PLC, Blalock JA, Borian FE (2005). Chronic depression: medication (Nefazodone) or Psychotherapy (CBASP) is effective when the other is not. Arch Gen Psychiatry.

[13] Health Quality Ontario (2016). Repetitive transcranial magnetic stimulation for treatment-resistant depression: a systematic review and meta-analysis of randomized controlled trials. Ont Health Technol Assess Ser.

[14] Barker AT, Jalinous R, Freeston IL (1985). Non-invasive magnetic stimulation of human motor cortex. Lancet.

[15] Bickford RG, Guidi M, Fortesque P, Swenson M (1987). Magnetic stimulation of human peripheral nerve and brain: response enhancement by combined magnetoelectrical technique. Neurosurgery.

[16] Pascual-Leone A, Houser CM, Reese K, Shotland LI, Grafman J, Sato S (1993). Safety of rapid-rate transcranial magnetic stimulation in normal volunteers. Electroencephalogr Clin Neurophysiol/Evoked Potentials Sect.

[17] Pascual-Leone A, Valls-Solé J, Wassermann EM, Hallett M (1994). Responses to rapid-rate transcranial magnetic stimulation of the human motor cortex. Brain.

[18] Wassermann EM, Lisanby SH (2001). Therapeutic application of repetitive transcranial magnetic stimulation: a review. Clin Neurophysiol.

[19] Pascual-Leone A, Rubio B, Pallardó F, Catalá MD (1996). Rapid-rate transcranial magnetic stimulation of left dorsolateral prefrontal cortex in drug-resistant depression. Lancet.

[20] Henriques JB, Davidson RI (1991). Left Frontal Hypoactivation in Depression.

[21] Allen JJB, Urry HL, Hitt SK, Coan JA (2004). The stability of resting frontal electroencephalographic asymmetry in depression. Psychophysiology.

[22] Baxter LR, Schwartz JM, Phelps ME, Mazziotta JC, Guze BH, Selin CE (1989). Reduction of Prefrontal Cortex Glucose Metabolism Common to Three Types of Depression. Arch Gen Psychiatry.

[23] Deslandes AC, de Moraes H, Pompeu FAMS, Ribeiro P, Cagy M, Capitão C (2008). Electroencephalographic frontal asymmetry and depressive symptoms in the elderly. Biol Psychol.

[24] Drevets WC. Functional anatomical abnormalities in limbic and prefrontal cortical structures in major depression. In: Progress in Brain Research. Elsevier; 2000. 413–31. (Cognition, emotion and autonomic responses: The integrative role of the prefrontal cortex and limbic structures; vol. 126). Cited 2022 Aug 18. Available from: https://www.sciencedirect.com/science/article/pii/S0079612300260275. 10.1016/S0079-6123(00)26027-5 11105660

[25] Kennedy SH, Evans KR, Krüger S, Mayberg HS, Meyer JH, McCann S (2001). Changes in regional brain glucose metabolism measured with positron emission tomography after paroxetine treatment of major depression. AJP.

[26] Orosz A, Jann K, Federspiel A, Horn H, Höfle O, Dierks T (2012). Reduced cerebral blood flow within the default-mode network and within total gray matter in major depression. Brain Connectivity.

[27] Ramasubbu R, Brown EC, Marcil LD, Talai AS, Forkert ND. Automatic Classification of Major Depression Disorder Using Arterial Spin Labeling MRI Perfusion Measurements. Psychiatry Clin Neurosci. 2019;73(8):486–93. 10.1111/pcn.12862 31077500

[28] Sackeim HA, Prohovnik I, Moeller JR, Brown RP, Apter S, Prudic J (1990). Regional cerebral blood flow in mood disorders: I. Comparison of major depressives and normal controls at rest. Arch General Psychiatry.

[29] Fitzgerald PB, Brown TL, Daskalakis ZJ (2002). The application of transcranial magnetic stimulation in psychiatry and neurosciences research: TMS in psychiatry and neurosciences. Acta Psychiatr Scand.

[30] Marangell LB, Martinez M, Jurdi RA, Zboyan H (2007). Neurostimulation therapies in depression: a review of new modalities. Acta Psychiatr Scand.

[31] Connolly KR, Helmer A, Cristancho MA, Cristancho P, O’Reardon JP (2012). Effectiveness of Transcranial Magnetic Stimulation in Clinical Practice Post-FDA Approval in the United States: Results Observed With the First 100 Consecutive Cases of Depression at an Academic Medical Center. J Clin Psychiatry.

[32] Brunoni AR, Chaimani A, Moffa AH, Razza LB, Gattaz WF, Daskalakis ZJ (2017). Repetitive transcranial magnetic stimulation for the acute treatment of major depressive episodes: a systematic review with network meta-analysis. JAMA Psychiat.

[33] Lefaucheur JP, Aleman A, Baeken C, Benninger DH, Brunelin J, Di Lazzaro V (2020). Evidence-based guidelines on the therapeutic use of repetitive transcranial magnetic stimulation (rTMS): An update (2014–2018). Clin Neurophysiol.

[34] Aaronson ST, Carpenter LL, Hutton TM, Kraus S, Mina M, Pages K (2022). Comparison of clinical outcomes with left unilateral and sequential bilateral Transcranial Magnetic Stimulation (TMS) treatment of major depressive disorder in a large patient registry. Brain Stimul.

[35] Larson J, Munkácsy E (2015). Theta-Burst LTP. Brain Res.

[36] Suppa A, Huang YZ, Funke K, Ridding MC, Cheeran B, Di Lazzaro V (2016). Ten years of theta burst stimulation in humans: established knowledge. Unknowns Prospects Brain Stimul.

[37] Blumberger DM, Vila-Rodriguez F, Thorpe KE, Feffer K, Noda Y, Giacobbe P (2018). Effectiveness of theta burst versus high-frequency repetitive transcranial magnetic stimulation in patients with depression (THREE-D): a randomised non-inferiority trial. Lancet.

[38] Blumberger DM, Mulsant BH, Thorpe KE, McClintock SM, Konstantinou GN, Lee HH (2022). Effectiveness of standard sequential bilateral repetitive transcranial magnetic stimulation vs bilateral theta burst stimulation in older adults with depression. JAMA Psychiat.

[39] Huang YZ, Edwards MJ, Rounis E, Bhatia KP, Rothwell JC (2005). Theta burst stimulation of the human motor cortex. Neuron.

[40] Huang YZ, Rothwell JC (2004). The effect of short-duration bursts of high-frequency, low-intensity transcranial magnetic stimulation on the human motor cortex. Clin Neurophysiol.

[41] Berlim MT, McGirr A, Rodrigues Dos Santos N, Tremblay S, Martins R (2017). Efficacy of theta burst stimulation (TBS) for major depression: an exploratory meta-analysis of randomized and sham-controlled trials. J Psychiatr Res.

[42] Plewnia C, Pasqualetti P, Große S, Schlipf S, Wasserka B, Zwissler B (2014). Treatment of major depression with bilateral theta burst stimulation: a randomized controlled pilot trial. J Affect Disord.

[43] Prasser J, Schecklmann M, Poeppl TB, Frank E, Kreuzer PM, Hajak G (2015). Bilateral prefrontal rTMS and theta burst TMS as an add-on treatment for depression: a randomized placebo controlled trial. World J Biol Psychiatry.

[44] Boyd LA, Hayward KS, Ward NS, Stinear CM, Rosso C, Fisher RJ (2017). Biomarkers of stroke recovery: Consensus-based core recommendations from the Stroke Recovery and Rehabilitation Roundtable. Int J Stroke.

[45] Fitzgerald P, Fountain S, Daskalakis Z (2006). A comprehensive review of the effects of rTMS on motor cortical excitability and inhibition. Clin Neurophysiol.

[46] Lefaucheur JP, André-Obadia N, Antal A, Ayache SS, Baeken C, Benninger DH (2014). Evidence-based guidelines on the therapeutic use of repetitive transcranial magnetic stimulation (rTMS). Clin Neurophysiol.

[47] Bajbouj M, Brakemeier EL, Schubert F, Lang UE, Neu P, Schindowski C (2005). Repetitive transcranial magnetic stimulation of the dorsolateral prefrontal cortex and cortical excitability in patients with major depressive disorder. Exp Neurol.

[48] Tremblay S, Rogasch NC, Premoli I, Blumberger DM, Casarotto S, Chen R (2019). Clinical utility and prospective of TMS–EEG. Clin Neurophysiol.

[49] Dhami P, Atluri S, Lee J, Knyahnytska Y, Croarkin PE, Blumberger DM (2021). Neurophysiological markers of response to theta burst stimulation in youth depression. Depress Anxiety.

[50] Voineskos D, Blumberger DM, Rogasch NC, Zomorrodi R, Farzan F, Foussias G, et al. Neurophysiological effects of repetitive transcranial magnetic stimulation (rTMS) in treatment resistant depression. Clin Neurophysiol. 2021;132(9):2306–16. 10.1016/j.clinph.2021.05.008 34167891

[51] Fox MD, Buckner RL, White MP, Greicius MD, Pascual-Leone A (2012). Efficacy of TMS targets for depression is related to intrinsic functional connectivity with the subgenual cingulate. Biol Psychiatry.

[52] Fox MD, Liu H, Pascual-Leone A (2013). Identification of reproducible individualized targets for treatment of depression with TMS based on intrinsic connectivity. Neuroimage.

[53] Fox MD, Halko MA, Eldaief MC, Pascual-Leone A (2012). Measuring and manipulating brain connectivity with resting state functional connectivity magnetic resonance imaging (fcMRI) and transcranial magnetic stimulation (TMS). Neuroimage.

[54] Weigand A, Horn A, Caballero R, Cooke D, Stern AP, Taylor SF (2018). Prospective validation that subgenual connectivity predicts antidepressant efficacy of transcranial magnetic stimulation sites. Biol Psychiatry.

[55] Baeken C, Lefaucheur JP, Van Schuerbeek P (2017). The impact of accelerated high frequency rTMS on brain neurochemicals in treatment-resistant depression: Insights from 1H MR spectroscopy. Clin Neurophysiol.

[56] Bhattacharyya P, Anand A, Lin J, Altinay M. Left Dorsolateral Prefrontal Cortex Glx/tCr Predicts Efficacy of High Frequency 4- to 6-Week rTMS Treatment and Is Associated With Symptom Improvement in Adults With Major Depressive Disorder: Findings From a Pilot Study. Frontiers in Psychiatry. 2021;12. Cited 2023 May 24. Available from: https://www.frontiersin.org/articles/10.3389/fpsyt.2021.665347. 10.3389/fpsyt.2021.665347 PMC8677827 34925079

[57] Mantovani A, Pavlicova M, Avery D, Nahas Z, McDonald WM, Wajdik CD (2012). Long-term efficacy of repeated daily prefrontal transcranial magnetic stimulation (TMS) in treatment-resistant depression. Depress Anxiety.

[58] Dunner DL, Aaronson ST, Sackeim HA, Janicak PG, Carpenter LL, Boyadjis T (2014). A multisite, naturalistic, observational study of transcranial magnetic stimulation for patients with Pharmacoresistant major depressive disorder: durability of benefit over a 1-year follow-up period. J Clin Psychiatry.

[59] Richieri R, Guedj E, Michel P, Loundou A, Auquier P, Lançon C (2013). Maintenance transcranial magnetic stimulation reduces depression relapse: a propensity-adjusted analysis. J Affect Disord.

[60] Lisanby SH, Sampson S, Husain MM, Petrides G, Knapp RG, McCall WV (2008). Toward individualized post-electroconvulsive therapy care: piloting the Symptom-Titrated, Algorithm-Based Longitudinal ECT (STABLE) intervention. J ECT.

[61] Chen J jun, Liu Z, Zhu D, Li Q, Zhang H, Huang H (2014). Bilateral vs. unilateral repetitive transcranial magnetic stimulation in treating major depression: a meta-analysis of randomized controlled trials. Psychiatry Res.

[62] Trevizol AP, Goldberger KW, Mulsant BH, Rajji TK, Downar J, Daskalakis ZJ (2019). Unilateral and bilateral repetitive transcranial magnetic stimulation for treatment-resistant late-life depression. Int J Geriatr Psychiatry.

[63] Ahmadizadeh MJ, Rezaei M (2018). Unilateral right and bilateral dorsolateral prefrontal cortex transcranial magnetic stimulation in treatment post-traumatic stress disorder: a randomized controlled study. Brain Res Bull.

[64] Voineskos D, Blumberger DM, Zomorrodi R, Rogasch NC, Farzan F, Foussias G (2019). Altered transcranial magnetic stimulation-electroencephalographic markers of inhibition and excitation in the dorsolateral prefrontal cortex in major depressive disorder. Biol Psychiat.

[65] Giacobbe P, Mithani K, Meng Y, Vila-Rodriguez F, Daskalakis ZJ, Downar J (2020). Evaluation of the effects of rTMS on self-reported quality of life and disability in treatment-resistant depression: a THREE-D study. J Affect Disord.

[66] Kaster TS, Downar J, Vila-Rodriguez F, Thorpe KE, Feffer K, Noda Y (2019). Trajectories of Response to dorsolateral prefrontal rTMS in major depression: A THREE-D study. Am J Psychiatry.

[67] Trevizol AP, Downar J, Vila-Rodriguez F, Thorpe KE, Daskalakis ZJ, Blumberger DM (2020). Predictors of remission after repetitive transcranial magnetic stimulation for the treatment of major depressive disorder: An analysis from the randomised non-inferiority THREE-D trial. EClinicalMedicine.

[68] Sheehan DV, Lecrubier Y, Sheehan KH, Amorim P, Janavs J, Weiller E, et al. The Mini-International Neuropsychiatric Interview (M.I.N.I.): the development and validation of a structured diagnostic psychiatric interview for DSM-IV and ICD-10. J Clin Psychiatry. 1998;59 Suppl 20:22-33;quiz 34–57. 9881538

[69] Sackeim HA (2001). The definition and meaning of treatment-resistant depression. J Clin Psychiatry.

[70] National Institutes of Health. NIMH Concomitant Medication Log Template. 2019. Cited 2023 Sep 19. Available from: https://www.nimh.nih.gov/sites/default/files/documents/funding/clinical-research/clinical-research-toolbox/documents/nimh_concomitant_medication_log_template_v1_july_2019.docx.

[71] Hamilton M (1960). A rating scale for depression. J Neurol Neurosurg Psychiatry.

[72] Montgomery SA, Åsberg M (1979). A new depression scale designed to be sensitive to change. Br J Psychiatry.

[73] Dantchev N, Widlöcher DJ (1998). The measurement of retardation in depression. J Clin Psychiatry.

[74] Young RC, Biggs JT, Ziegler VE, Meyer DA (1978). A rating scale for mania: reliability, validity and sensitivity. Br J Psychiatry.

[75] Lukasiewicz M, Gerard S, Besnard A, Falissard B, Perrin E, Sapin H (2013). Young Mania Rating Scale: how to interpret the numbers? Determination of a severity threshold and of the minimal clinically significant difference in the EMBLEM cohort. Int J Methods Psychiatr Res.

[76] Posner K, Brown GK, Stanley B, Brent DA, Yershova KV, Oquendo MA (2011). The Columbia-suicide severity rating scale: initial validity and internal consistency findings from three multisite studies with adolescents and adults. AJP.

[77] Folstein MF, Folstein SE, McHugh PR (1975). “Mini-mental state”: a practical method for grading the cognitive state of patients for the clinician. J Psychiatr Res.

[78] Rush AJ, Trivedi MH, Ibrahim HM, Carmody TJ, Arnow B, Klein DN (2003). The 16-Item quick inventory of depressive symptomatology (QIDS), clinician rating (QIDS-C), and self-report (QIDS-SR): a psychometric evaluation in patients with chronic major depression. Biol Psychiat.

[79] Altman EG, Hedeker D, Peterson JL, Davis JM (1997). The Altman self-rating mania scale. Biol Psychiat.

[80] Beck AT, Epstein N, Brown G, Steer RA (1988). An inventory for measuring clinical anxiety: Psychometric properties. J Consult Clin Psychol.

[81] Beck AT, Steer RA, Ranieri WF (1988). Scale for suicide ideation: Psychometric properties of a self-report version. J Clin Psychol.

[82] Stevanovic D (2011). Quality of Life Enjoyment and Satisfaction Questionnaire-short form for quality of life assessments in clinical practice: a psychometric study. J Psychiatr Ment Health Nurs.

[83] Shah N, Cader M, Andrews B, McCabe R, Stewart-Brown SL (2021). Short Warwick-Edinburgh Mental Well-being Scale (SWEMWBS): performance in a clinical sample in relation to PHQ-9 and GAD-7. Health Qual Life Outcomes.

[84] Buysse DJ, Reynolds CF, Monk TH, Berman SR, Kupfer DJ (1989). The Pittsburgh sleep quality index: a new instrument for psychiatric practice and research. Psychiatry Res.

[85] Parrott AC, Hindmarch I (1978). Factor analysis of a sleep evaluation questionnaire. Psychol Med.

[86] Blevins CA, Weathers FW, Davis MT, Witte TK, Domino JL (2015). The Posttraumatic Stress Disorder Checklist for DSM-5 (PCL-5): Development and Initial Psychometric Evaluation. J Trauma Stress.

[87] Hoddes E, Dement W, Zarcone V, Hoddes E, Dement W, Zarcone V (1972). The development and use of the Stanford sleepiness scale (SSS). Psychophysiology..

[88] Lee PH, Macfarlane DJ, Lam T, Stewart SM (2011). Validity of the international physical activity questionnaire short form (IPAQ-SF): A systematic review. Int J Behav Nutr Phys Act.

[89] Cotovio G, Boes AD, Press DZ, Oliveira-Maia AJ, Pascual-Leone A (2022). In older adults the antidepressant effect of repetitive transcranial magnetic stimulation is similar but occurs later than in younger adults. Front Aging Neurosci.

[90] Rossi S, Antal A, Bestmann S, Bikson M, Brewer C, Brockmöller J (2021). Safety and recommendations for TMS use in healthy subjects and patient populations, with updates on training, ethical and regulatory issues: Expert Guidelines. Clin Neurophysiol.

[91] Kennedy SH, Milev R, Giacobbe P, Ramasubbu R, Lam RW, Parikh SV (2009). Canadian Network for Mood and Anxiety Treatments (CANMAT) Clinical guidelines for the management of major depressive disorder in adults. J Affect Disord.

[92] Milev RV, Giacobbe P, Kennedy SH, Blumberger DM, Daskalakis ZJ, Downar J (2016). Canadian Network for Mood and Anxiety Treatments (CANMAT) 2016 Clinical Guidelines for the Management of Adults with Major Depressive Disorder: Section 4. Neurostimulation Treatments. Can J Psychiatry..

[93] Mescher M, Merkle H, Kirsch J, Garwood M, Gruetter R (1998). Simultaneous in vivo spectral editing and water suppression. NMR Biomed.

[94] Narayan GA, Hill KR, Wengler K, He X, Wang J, Yang J (2022). Does the change in glutamate to gaba ratio correlate with change in depression severity? A randomized double-blind clinical trial. Mol Psychiatry.

[95] Wu X, Han S, Yang Y, Dai H, Wu P, Zhao H (2022). Decreased Brain GABA Levels in Patients with Migraine Without Aura: An Exploratory Proton Magnetic Resonance Spectroscopy Study. Neuroscience.

[96] Provencher SW (1993). Estimation of metabolite concentrations from localized in vivo proton NMR spectra. Magn Reson Med.

[97] Rossini PM, Burke D, Chen R, Cohen LG, Daskalakis Z, Di Iorio R (2015). Non-invasive electrical and magnetic stimulation of the brain, spinal cord, roots and peripheral nerves: Basic principles and procedures for routine clinical and research application. An updated report from an I.F.C.N Committee. Clin Neurophysiol.

[98] Sekiguchi H, Takeuchi S, Kadota H, Kohno Y, Nakajima Y (2011). TMS-induced artifacts on EEG can be reduced by rearrangement of the electrode’s lead wire before recording. Clin Neurophysiol.

[99] Rocchi L, Di Santo A, Brown K, Ibáñez J, Casula E, Rawji V (2021). Disentangling EEG responses to TMS due to cortical and peripheral activations. Brain Stimul.

[100] Delorme A, Makeig S (2004). EEGLAB: an open source toolbox for analysis of single-trial EEG dynamics including independent component analysis. J Neurosci Methods.

[101] Oostenveld R, Fries P, Maris E, Schoffelen JM. FieldTrip: Open source software for advanced analysis of MEG, EEG, and invasive electrophysiological data. Comput Intell Neurosci. 2011;2011:156869. 10.1155/2011/156869 PMC3021840 21253357

[102] Desforges M, Hadas I, Mihov B, Morin Y, Braün M, Lioumis P (2022). Dose-response of intermittent theta burst stimulation of the prefrontal cortex: A TMS-EEG study. Clin Neurophysiol.

[103] Dziura JD, Post LA, Zhao Q, Fu Z, Peduzzi P (2013). Strategies for dealing with missing data in clinical trials: from design to analysis. Yale J Biol Med.

[104] Sterne JAC, White IR, Carlin JB, Spratt M, Royston P, Kenward MG (2009). Multiple imputation for missing data in epidemiological and clinical research: potential and pitfalls. BMJ.

[105] Harris PA, Taylor R, Minor BL, Elliott V, Fernandez M, O’Neal L (2019). The REDCap consortium: Building an international community of software platform partners. J Biomed Inform.

[106] Li CT, Chen MH, Juan CH, Huang HH, Chen LF, Hsieh JC (2014). Efficacy of prefrontal theta-burst stimulation in refractory depression: a randomized sham-controlled study. Brain.

[107] Faul F, Erdfelder E, Lang AG, Buchner A (2007). G*Power 3: A flexible statistical power analysis program for the social, behavioral, and biomedical sciences. Behav Res Methods.

[108] Cohen J (1988). Statistical Power Analysis for the Behavioral Sciences.

[109] Fitzgerald PB (2020). An update on the clinical use of repetitive transcranial magnetic stimulation in the treatment of depression. J Affect Disord.

[110] Rachid F (2018). Maintenance repetitive transcranial magnetic stimulation (rTMS) for relapse prevention in with depression: a review. Psychiatry Res.

[111] Fitzgerald PB (2019). Is maintenance repetitive transcranial magnetic stimulation for patients with depression a valid therapeutic strategy?. Clin Pharmacol Ther.

[112] Sagar R, Pattanayak RD (2017). Potential biomarkers for bipolar disorder: Where do we stand?. Indian J Med Res.

[113] Wichniak A, Wierzbicka A, Jernajczyk W (2013). Sleep as a biomarker for depression. Int Rev Psychiatry.

[114] Lam RW, Chan P, Wilkins-Ho M, Yatham LN (2008). Repetitive transcranial magnetic stimulation for treatment-resistant depression: a systematic review and metaanalysis. Can J Psychiatry.

[115] O’Reardon JP, Solvason HB, Janicak PG, Sampson S, Isenberg KE, Nahas Z (2007). Efficacy and safety of transcranial magnetic stimulation in the acute treatment of major depression: a multisite randomized controlled trial. Biol Psychiat.

[116] Chen R, Berardelli A, Bhattacharya A, Bologna M, Chen KHS, Fasano A (2022). Clinical neurophysiology of Parkinson’s disease and parkinsonism. Clin Neurophysiol Pract.

[117] Chou Y hui, Sundman M, Ton That V, Green J, Trapani C (2022). Cortical excitability and plasticity in Alzheimer’s disease and mild cognitive impairment: A systematic review and meta-analysis of transcranial magnetic stimulation studies. Ageing Res Rev.

[118] Citri A, Malenka RC (2008). Synaptic Plasticity: Multiple Forms, Functions, and Mechanisms. Neuropsychopharmacol.

[119] Chaves AR, Snow NJ, Alcock LR, Ploughman M (2021). Probing the brain-body connection using Transcranial Magnetic Stimulation (TMS): validating a promising tool to provide biomarkers of neuroplasticity and central nervous system function. Brain Sci.

[120] Civardi C, Boccagni C, Vicentini R, Bolamperti L, Tarletti R, Varrasi C (2001). Cortical excitability and sleep deprivation: a transcranial magnetic stimulation study. J Neurol Neurosurg Psychiatry.

[121] Cortese F, Coppola G, Di Lenola D, Serrao M, Di Lorenzo C, Parisi V (2017). Excitability of the motor cortex in patients with migraine changes with the time elapsed from the last attack. J Headache Pain.

[122] Nanda S, Arya S, Tiwari V, Srikumar V, Kumar U, Bhatia R (2019). Transcranial Magnetic Stimulation (TMS) induced Motor Evoked Potential (MEP) in Chronic Pain Patients. Brain Stimul.

[123] Sundman MH, Lim K, Ton That V, Mizell JM, Ugonna C, Rodriguez R (2020). Transcranial magnetic stimulation reveals diminished homoeostatic metaplasticity in cognitively impaired adults. Brain Commun..

[124] Yuksel H, Topalkara KK (2021). Increased cortical excitability in female migraineurs: a transcranial magnetic stimulation study conducted in the preovulatory phase. J Clin Neurol.

[125] Aloizou AM, Pateraki G, Anargyros K, Siokas V, Bakirtzis C, Liampas I (2021). Transcranial magnetic stimulation (TMS) and repetitive TMS in multiple sclerosis. Rev Neurosci.

[126] Saini M, Singh N, Kumar N, Srivastava MVP, Mehndiratta A. A novel perspective of associativity of upper limb motor impairment and cortical excitability in sub-acute and chronic stroke. Front Neurosci. 2022;16. Cited 2022 Aug 23. Available from: https://www.frontiersin.org/articles/10.3389/fnins.2022.832121. 10.3389/fnins.2022.832121 PMC9358254 35958985

[127] Suzuki YI, Shibuya K, Misawa S, Suichi T, Tsuneyama A, Kojima Y, et al. Relationship between motor cortical and peripheral axonal hyperexcitability in amyotrophic lateral sclerosis. J Neurol Neurosurg Psychiatry. 2022. 10.1136/jnnp-2021-328550. 10.1136/jnnp-2021-328550 35995552

[128] Cantone M, Bramanti A, Lanza G, Pennisi M, Bramanti P, Pennisi G (2017). Cortical plasticity in depression: a neurochemical perspective from transcranial magnetic stimulation. ASN Neuro.

[129] Fitzgerald PB, Brown TL, Marston NAU, Daskalakis ZJ, de Castella A, Bradshaw JL (2004). Motor cortical excitability and clinical response to rTMS in depression. J Affect Disord.

[130] Veronezi BP, Moffa AH, Carvalho AF, Galhardoni R, Simis M, Benseñor IM (2016). Evidence for increased motor cortical facilitation and decreased inhibition in atypical depression. Acta Psychiatr Scand.

[131] Bajbouj M, Lisanby SH, Lang UE, Danker-Hopfe H, Heuser I, Neu P (2006). Evidence for impaired cortical inhibition in patients with unipolar major depression. Biol Psychiat.

[132] Lefaucheur JP, Lucas B, Andraud F, Hogrel JY, Bellivier F, Del Cul A (2008). Inter-hemispheric asymmetry of motor corticospinal excitability in major depression studied by transcranial magnetic stimulation. J Psychiatr Res.

[133] Levinson AJ, Fitzgerald PB, Favalli G, Blumberger DM, Daigle M, Daskalakis ZJ (2010). Evidence of cortical inhibitory deficits in major depressive disorder. Biol Psychiat.

[134] Ge R, Humaira A, Gregory E, Alamian G, MacMillan EL, Barlow L (2022). Predictive value of acute neuroplastic response to rTMS in treatment outcome in depression: a concurrent TMS-fMRI Trial. Am J Psychiatry.

[135] Strafella R, Momi D, Zomorrodi R, Lissemore J, Noda Y, Chen R (2023). Identifying Neurophysiological Markers of Intermittent Theta-Burst Stimulation in Treatment-Resistant Depression using Transcranial Magnetic Stimulation- Electroencephalography. Biol Psychiatry.

[136] Javaheripour N, Li M, Chand T, Krug A, Kircher T, Dannlowski U (2021). Altered resting-state functional connectome in major depressive disorder: a mega-analysis from the PsyMRI consortium. Transl Psychiatry.

[137] Rao NP, Venkatasubramanian G, Gangadhar BN (2011). Proton magnetic resonance spectroscopy in depression. Indian J Psychiatry.

[138] Cole EJ, Stimpson KH, Bentzley BS, Gulser M, Cherian K, Tischler C (2020). Stanford accelerated intelligent neuromodulation therapy for treatment-resistant depression. Am J Psychiatry.

[139] Chung SW, Rogasch NC, Hoy KE, Sullivan CM, Cash RFH, Fitzgerald PB (2017). Impact of different intensities of intermittent theta burst stimulation on the cortical properties during TMS-EEG and working memory performance. Hum Brain Mapp.

