# Supplementary material for: A naturalistic study comparing the efficacy of unilateral and bilateral sequential theta burst stimulation in treating major depression – the U-B-D study protocol
Source: BMC Psychiatry. 2023 Oct 10;23:739. doi: 10.1186/s12888-023-05243-4 (PMC10566125; doi:10.1186/s12888-023-05243-4)
Supplement: Supplementary file 1 — Additional file 1: Appendix I. World Health Organization Trial Registration Data Set. Appendix II. SPIRIT guidelines: REB Revision Chronology. Appendix III. SPIRIT 2013 Checklist: Recommended items to address in a clinical trial protocol and related documents*. Appendix IV. Informed Consent Form for Participation in a Research Study. [file 12888_2023_5243_MOESM1_ESM.docx]

**APPENDIX I**

World Health Organization Trial Registration Data Set

| Data Category | Information |
| --- | --- |
| Primary registry and trial identification number | ClinicalTrials.gov  #NCT04142996 |
| Date of registration in primary registry | October 15, 2019 |
| Secondary identifying numbers | ID: 2019017 |
| Source(s) of monetary or material support | Emerging Research Innovators in Mental Health (eRIMh) |
| Primary sponsor | Royal Ottawa Mental Health Centre  Donation obtained through the Royal Ottawa Foundation and the Ottawa Community Foundation  Research Scholar Junior 1 grant (#297133) from the Fonds de Recherche en Santé – Québec (FRQ-S) |
| Secondary sponsor(s) |  |
| Contact for public queries | Sara Tremblay  sara.tremblay@theroyal.ca |
| Contact for scientific queries | Sara Tremblay  sara.tremblay@theroyal.ca |
| Public title | Comparing Uni- and Bi-lateral TBS in Major Depression |
| Scientific title | A Naturalistic Study Comparing the Efficacy of Unilateral and Bilateral Sequential Theta Burst Stimulation in Treating Major Depression –The U-B-D Study |
| Countries of recruitment | Canada |
| Health condition(s) or problem(s) studied | Treatment resistant depression |
| Interventions | Uni-and bilateral theta burst stimulation (TBS) |
| Key inclusion and exclusion criteria | Inclusion criteria: ≥ 18 years-old, biological male or female, primary and/or predominant diagnosis of MDE without psychotic features  Exclusion criteria: current or past substance abuse, neurological illness, acute suicidality, have a contraindication to transcranial magnetic stimulation (TMS) |
| Study type | Double-blinded randomized, naturalistic, non-inferiority trial |
| Date of first enrolment | December 12, 2019 |
| Target sample size | 256 |
| Recruitment status | Ongoing recruitment |
| Primary outcome(s) | Efficacy of bilateral sequential TBS and unilateral TBS on reducing depressive symptoms as per HRSD-17. |
| Key secondary outcomes | Clinical Neuropsychological Assessments, TMS-EEG, MRI and MRS, and Single and Paired-Pulse TMS. |

**APPENDIX II**

**SPIRIT guidelines: REB Revision Chronology**

| ***Protocol Version & Date*** | ***Amendment Description*** |
| --- | --- |
| *Version 1: 10-Jun-2019* | Original |
| *Version 2: 02-Aug-2019* | Revisions from initial REB review (i.e. letter of concerns). |
| *Version 3: 16-Aug-2019* | Minor typographical and grammatical changes. |
| *Version 4: 09-Dec-2019* | Clarifications to screening visit, and addition of clinical scale (MADRS) for secondary outcome measure. |
| *Version 5: 10-Dec-2019* | Revised compensation, and treatment intensity based on established guidelines for rTMS research. |
| *Version 6: 11-Mar-2020* | Revised schedule of events to ease participant burden and allow for more flexibility at screening and consultation visits. |
| *Version 7:* *02-Jun-2021* | Addition of MRI visit to allow for more precise treatment targeting. Clarifications made to exclusion criteria and compensation. |
| *Version 8: 23-Sep-2021* | Addition of co-investigator. |
| *Version 9: 31-Oct-2021* | Revised maintenance phase of the trial to a flexible schedule only (instead of fixed), which reduced the number of visits during the Covid-19 pandemic. Added co-investigator and an additional questionnaire (Stanford Sleepiness Scale). |
| *Version 10: 25-Nov-2021* | Study contact information changed for new staff member. |
| *Version 11: 24-Jan-2022* | Increased frequency of follow-ups with study psychiatrist to ensure patient safety and well-being. |
| *Version 12: 27-Apr-2022* | Translated all study documents to French for more accessibility. |
| *Version 13: 14-Jul-2022* | Revised clinical assessment schedule to add another visit during treatment course, and added another questionnaire to monitor physical activity. Revised follow-ups with study psychiatrist in maintenance phase on an as-needed basis for convenience. |
| *Version 14: 7-Sep-2022* | Added option to discharge patients in the maintenance phase at discretion of study doctors if clinical scores go back to baseline. |
| *Version 15: 16-Jan-2023* | Addition of withdrawal/discontinuation criteria. Revised schedule of events and when the consultation/follow-ups with study psychiatrist will take place. |
| *Version 16: 4-Apr-2023* | Clarifications made to the inclusion/exclusion criteria and discontinuation criteria in the maintenance phase. |

**APPENDIX III**


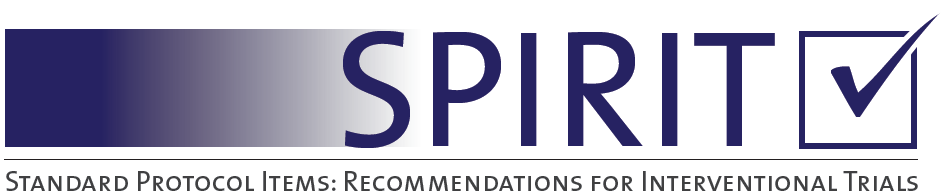


SPIRIT 2013 Checklist: Recommended items to address in a clinical trial protocol and related documents*

| Section/item | Item No | Description | Addressed on page number |
| --- | --- | --- | --- |
| **Administrative information** | | |  |
| Title | 1 | Descriptive title identifying the study design, population, interventions, and, if applicable, trial acronym | Title page |
| Trial registration | 2a | Trial identifier and registry name. If not yet registered, name of intended registry | Abstract page, Appendix I |
|  | 2b | All items from the World Health Organization Trial Registration Data Set | Appendix I |
| Protocol version | 3 | Date and version identifier | Appendix II |
| Funding | 4 | Sources and types of financial, material, and other support | Section “Funding”, Appendix I |
| Roles and responsibilities | 5a | Names, affiliations, and roles of protocol contributors | Section “Authors’ Contributions”, Title page |
|  | 5b | Name and contact information for the trial sponsor | Appendix I |
|  | 5c | Role of study sponsor and funders, if any, in study design; collection, management, analysis, and interpretation of data; writing of the report; and the decision to submit the report for publication, including whether they will have ultimate authority over any of these activities | Section “Authors’ Contributions” |
|  | 5d | Composition, roles, and responsibilities of the coordinating centre, steering committee, endpoint adjudication committee, data management team, and other individuals or groups overseeing the trial, if applicable (see Item 21a for data monitoring committee) | Not applicable (see item 21a) |
| Introduction |  |  |  |
| Background and rationale | 6a | Description of research question and justification for undertaking the trial, including summary of relevant studies (published and unpublished) examining benefits and harms for each intervention | Introduction |
|  | 6b | Explanation for choice of comparators | Introduction |
| Objectives | 7 | Specific objectives or hypotheses | Introduction |
| Trial design | 8 | Description of trial design including type of trial (eg, parallel group, crossover, factorial, single group), allocation ratio, and framework (eg, superiority, equivalence, noninferiority, exploratory) | Introduction |
| Methods: Participants, interventions, and outcomes | | |  |
| Study setting | 9 | Description of study settings (eg, community clinic, academic hospital) and list of countries where data will be collected. Reference to where list of study sites can be obtained | Section 2.2, Appendix I |
| Eligibility criteria | 10 | Inclusion and exclusion criteria for participants. If applicable, eligibility criteria for study centres and individuals who will perform the interventions (eg, surgeons, psychotherapists) | Table 2 |
| Interventions | 11a | Interventions for each group with sufficient detail to allow replication, including how and when they will be administered | Methods section |
|  | 11b | Criteria for discontinuing or modifying allocated interventions for a given trial participant (eg, drug dose change in response to harms, participant request, or improving/worsening disease) | Section 2.1 |
|  | 11c | Strategies to improve adherence to intervention protocols, and any procedures for monitoring adherence (eg, drug tablet return, laboratory tests) | Section 2.3 |
|  | 11d | Relevant concomitant care and interventions that are permitted or prohibited during the trial | Table 2 |
| Outcomes | 12 | Primary, secondary, and other outcomes, including the specific measurement variable (eg, systolic blood pressure), analysis metric (eg, change from baseline, final value, time to event), method of aggregation (eg, median, proportion), and time point for each outcome. Explanation of the clinical relevance of chosen efficacy and harm outcomes is strongly recommended | Table 1 |
| Participant timeline | 13 | Time schedule of enrolment, interventions (including any run-ins and washouts), assessments, and visits for participants. A schematic diagram is highly recommended (see Figure) | Figure 2 |
| Sample size | 14 | Estimated number of participants needed to achieve study objectives and how it was determined, including clinical and statistical assumptions supporting any sample size calculations | Section 3.2, Appendix I |
| Recruitment | 15 | Strategies for achieving adequate participant enrolment to reach target sample size | Sections 2.3 and 3.2 |
| **Methods: Assignment of interventions (for controlled trials)** | | |  |
| Allocation: |  |  |  |
| Sequence generation | 16a | Method of generating the allocation sequence (eg, computer-generated random numbers), and list of any factors for stratification. To reduce predictability of a random sequence, details of any planned restriction (eg, blocking) should be provided in a separate document that is unavailable to those who enrol participants or assign interventions | Section 2.4 |
| Allocation concealment mechanism | 16b | Mechanism of implementing the allocation sequence (eg, central telephone; sequentially numbered, opaque, sealed envelopes), describing any steps to conceal the sequence until interventions are assigned | Section 2.4 |
| Implementation | 16c | Who will generate the allocation sequence, who will enrol participants, and who will assign participants to interventions | Section 2.4 |
| Blinding (masking) | 17a | Who will be blinded after assignment to interventions (eg, trial participants, care providers, outcome assessors, data analysts), and how | Sections 2.4 and 2.5 |
|  | 17b | If blinded, circumstances under which unblinding is permissible, and procedure for revealing a participant’s allocated intervention during the trial | Sections 2.4 and 2.5 |
| **Methods: Data collection, management, and analysis** | | |  |
| Data collection methods | 18a | Plans for assessment and collection of outcome, baseline, and other trial data, including any related processes to promote data quality (eg, duplicate measurements, training of assessors) and a description of study instruments (eg, questionnaires, laboratory tests) along with their reliability and validity, if known. Reference to where data collection forms can be found, if not in the protocol | See Table 3 for references and Section 2.6.1 |
|  | 18b | Plans to promote participant retention and complete follow-up, including list of any outcome data to be collected for participants who discontinue or deviate from intervention protocols | Section 2.3 |
| Data management | 19 | Plans for data entry, coding, security, and storage, including any related processes to promote data quality (eg, double data entry; range checks for data values). Reference to where details of data management procedures can be found, if not in the protocol | Section 3.1 |
| Statistical methods | 20a | Statistical methods for analysing primary and secondary outcomes. Reference to where other details of the statistical analysis plan can be found, if not in the protocol | Sections 3.3 to 3.5 |
|  | 20b | Methods for any additional analyses (eg, subgroup and adjusted analyses) | Sections 3.3 to 3.5 |
|  | 20c | Definition of analysis population relating to protocol non-adherence (eg, as randomised analysis), and any statistical methods to handle missing data (eg, multiple imputation) | Sections 3, 3.3. and 3.4 |
| **Methods: Monitoring** | | |  |
| Data monitoring | 21a | Composition of data monitoring committee (DMC); summary of its role and reporting structure; statement of whether it is independent from the sponsor and competing interests; and reference to where further details about its charter can be found, if not in the protocol. Alternatively, an explanation of why a DMC is not needed | DMC is not needed as per the Canada Tri-Council Policy Statement: Ethical Conduct for Research Involving Humans (TCPS 2) |
|  | 21b | Description of any interim analyses and stopping guidelines, including who will have access to these interim results and make the final decision to terminate the trial | Section 2.1 and 3.5 |
| Harms | 22 | Plans for collecting, assessing, reporting, and managing solicited and spontaneously reported adverse events and other unintended effects of trial interventions or trial conduct | Section 2.5 |
| Auditing | 23 | Frequency and procedures for auditing trial conduct, if any, and whether the process will be independent from investigators and the sponsor | Section 3.1 |
| Ethics and dissemination | | |  |
| Research ethics approval | 24 | Plans for seeking research ethics committee/institutional review board (REC/IRB) approval | Section 2.2 |
| Protocol amendments | 25 | Plans for communicating important protocol modifications (eg, changes to eligibility criteria, outcomes, analyses) to relevant parties (eg, investigators, REC/IRBs, trial participants, trial registries, journals, regulators) | Section 2.2 |
| Consent or assent | 26a | Who will obtain informed consent or assent from potential trial participants or authorised surrogates, and how (see Item 32) | Section 2.1 |
|  | 26b | Additional consent provisions for collection and use of participant data and biological specimens in ancillary studies, if applicable | Not applicable |
| Confidentiality | 27 | How personal information about potential and enrolled participants will be collected, shared, and maintained in order to protect confidentiality before, during, and after the trial | Section 3.1 |
| Declaration of interests | 28 | Financial and other competing interests for principal investigators for the overall trial and each study site | Section ”Competing Interests" |
| Access to data | 29 | Statement of who will have access to the final trial dataset, and disclosure of contractual agreements that limit such access for investigators | Section ”Availability of Data and Materials" |
| Ancillary and post-trial care | 30 | Provisions, if any, for ancillary and post-trial care, and for compensation to those who suffer harm from trial participation | Section 2.1 |
| Dissemination policy | 31a | Plans for investigators and sponsor to communicate trial results to participants, healthcare professionals, the public, and other relevant groups (eg, via publication, reporting in results databases, or other data sharing arrangements), including any publication restrictions | Section 3.1 |
|  | 31b | Authorship eligibility guidelines and any intended use of professional writers | Section ”Authors’ contributions" |
|  | 31c | Plans, if any, for granting public access to the full protocol, participant-level dataset, and statistical code | Section ”Availability of Data and Materials" |
| Appendices |  |  |  |
| Informed consent materials | 32 | Model consent form and other related documentation given to participants and authorised surrogates | Appendix IV |
| Biological specimens | 33 | Plans for collection, laboratory evaluation, and storage of biological specimens for genetic or molecular analysis in the current trial and for future use in ancillary studies, if applicable | Not applicable |

*It is strongly recommended that this checklist be read in conjunction with the SPIRIT 2013 Explanation & Elaboration for important clarification on the items. Amendments to the protocol should be tracked and dated. The SPIRIT checklist is copyrighted by the SPIRIT Group under the Creative Commons “[Attribution-NonCommercial-NoDerivs 3.0 Unported](http://www.creativecommons.org/licenses/by-nc-nd/3.0/)” license.

**APPENDIX IV**


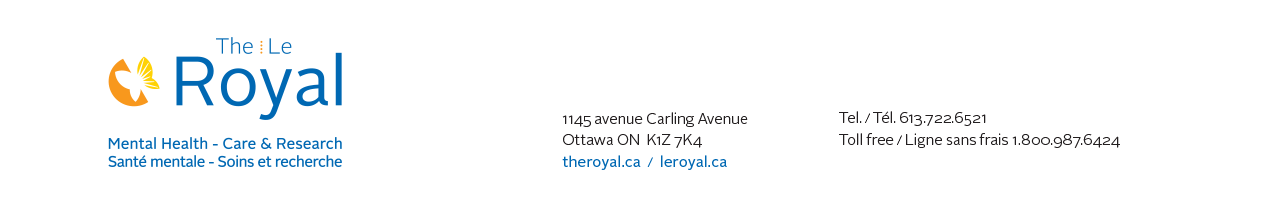


**Informed Consent Form for Participation in a Research Study**

**Study Title**: A naturalistic study comparing the efficacy of uni- and bi-lateral theta burst stimulation major depression (REB #2019017)

**Study Doctor**: Sara Tremblay, Neuromodulation Unit, 613-722-6521 ext. 6227

**Funder(s):** Emerging Researcher in Mental Health (eRIMh) Fund

INTRODUCTION

You are being invited to participate in a clinical trial (a type of study that involves research). You are invited to participate in this trial because you have a diagnosis of Major Depressive Episode and are interested in pursuing treatment using Repetitive Transcranial Magnetic Stimulation (rTMS).This consent form provides you with information to help you make an informed choice. Please read this document carefully and ask any questions you may have. All your questions should be answered to your satisfaction before you decide whether to participate in this research study. Please take your time in making your decision. You may find it helpful to discuss it with your friends and family.

Taking part in this study is voluntary. Deciding not to take part or deciding to leave the study later will not result in any penalty or affect current or future health care.

IS THERE A CONFLICT OF INTEREST?

There are no conflicts of interest to declare related to this study.

WHAT IS THE BACKGROUND INFORMATION FOR THIS STUDY?

The standard or usual treatment for depression is medication and/or psychotherapy. Alternatively, rTMS has been used as a treatment for depression for the past two decades and is now part of treatment recommendation for depression. Health Canada, the regulatory body that oversees the use of medical devices in Canada, has approved the use of rTMS in treating depression since 2002.

rTMS is a tool that uses magnetic fields to activate a specific region of the brain. These magnetic fields are produced by a small stimulating coil, which is made of loops of copper wires covered with plastic that resemble a big spoon. The coil is positioned firmly but comfortably against the head. Over time, the magnetic field pulses can gradually change the activity level of the stimulated brain region and improve symptoms of depression. The standard rTMS treatment consists of 30 to 45 minutes daily sessions (on weekdays only), applied over 4 to 6 weeks. Typically, half of people receiving treatment will respond favorably (which is similar to medication), and effects will last up to 12 months after the last treatment.

There have been some recent advances in rTMS devices that now allows having the same efficacy, using daily sessions that last 4 minutes (10 times shorter). This new technique is called theta burst stimulation (TBS). We can apply TBS to one side or the brain (unilateral) or both sides of the brain (bilateral). We are unsure which type of stimulation is most efficient for reducing symptoms of depression.

In addition, since effects typically last up to 12 months after treatment, the standard of care with rTMS is to provide a maintenance phase after the end of treatment in which the frequency of rTMS sessions is gradually decreased. There is no clear recommendation as to what schedule of maintenance to adopt. Typically, maintenance is offered for up to 6 months. We do not know if it is better to have a fixed schedule in which we determine a fixed number of sessions at a specific frequency, or a flexible schedule in which a treatment will be offered only if there is a change in symptoms.

WHY IS THIS STUDY BEING DONE?

The first purpose of this study is to determine which technique is most effective to reduce symptoms of depression amongst the unilateral and bilateral TBS conditions. To do so, all participants will receive bilateral stimulation. However, the unilateral group will receive placebo stimulation on one side of the brain. This is used to make the study more reliable. This study will also allow us to determine if how the brain activity is modified after one TBS session can predict if someone is more likely to benefit from treatment.

The second purpose of this study is to determine if the flexible maintenance protocol is efficient in maintaining benefits from TBS treatment compared to no maintenance phase.

WHAT OTHER CHOICES ARE THERE?

You do not have to take part in this study in order to receive treatment or care. Other options (in addition to the standard or usual treatment described above) may include, but are not limited to:

- treatment prescribed by your referring physician (e.g. antidepressant, psychotherapy)
- other research studies may be available if you do not take part in this study

Please talk to your usual doctor or the study doctor about the known benefits and risks of these other options before you decide to take part in this study. Your usual doctor or the study doctor can also discuss with you what will happen if you decide not to undertake any treatment at this time.

HOW MANY PEOPLE WILL TAKE PART IN THIS STUDY?

It is anticipated that about 256 people will take part in this study at the Royal Ottawa Mental Health Centre, having been referred by their treating physician from the ROMHC or from a medical clinic in the Ottawa/Gatineau region.

This study should take 4 years to complete and the results should be known in about 5 years.

WHAT WILL HAPPEN DURING THIS STUDY?

ASSIGNMENT TO A GROUP
If you decide to participate, you will be randomly assigned to one of the two treatment conditions. Randomization means that you are put into a group by chance (like flipping a coin). There is no way to predict which group you will be assigned to. You will have a 1 in 2 chance of being placed in either group. Neither you, the study staff, or the study doctors can choose what group you will be in.

This is a double-blind study, which means that neither you, the study doctors, the study staff, or your usual health care providers will know which group you are in. Your group assignment can be identified if medically necessary. Requests to reveal your assignment for your information or participation in other research studies will not be considered until this study has been completed and the results are known.

It is possible that you may finish the screening phase and be ready to enter the treatment phase of the study, but not be enrolled (or accepted) into the study.

If you benefit from treatment, you will be assigned to one of the two maintenance conditions: either fixed or flexible schedule. You will be told which maintenance condition you are in. However, your TBS condition will remain blinded.

WHAT IS THE STUDY INTERVENTION?

If you agree to take part in this study, you will receive TBS treatment at the ROMCH for 5 days a week over the course of 4-6 weeks, for a total of 20-30 sessions. Sessions will run on weekdays only.

Group 1: Unilateral TBS

If you are randomized to this group, TBS will be applied to your left forehead region followed by a placebo stimulation of your right forehead region, for a total duration of 4 minutes.

Group 2: Bilateral TBS

If you are randomized to this group, TBS will be applied to your left forehead region followed by stimulation of your right forehead region, for a total duration of 4 minutes.

If you benefit from the treatment, you will then move on to the maintenance phase in which you will receive the same TBS condition as for the treatment, over a period of six months at a gradually decreasing frequency.

Maintenance: Flexible Schedule

If you are randomized to this group, TBS will be applied twice a week for one month and according to your symptoms for the remaining 5 months.

WHAT ELSE DO I NEED TO KNOW ABOUT THE STUDY INTERVENTION?

If you have side effects while you are on this study, the study doctor may make changes to the intervention. You will be instructed to remain on your current psychiatric medication or psychotherapy as instructed by your physician

WHAT ARE THE STUDY PROCEDURES?

*Non-Experimental Procedures*

The following test will be done as part of your standard care and results will not be used as data in the study:

- **Urine sample:** urine will be collected *at* baseline*.* This urine sample will be sent to the laboratory at the ROMHC where they will be examined for drug consumption and pregnancy (female only).

The following tests will be done as part of this study. Some of these tests may be done more frequently than if you were not taking part in this study and some may be done only for the purpose of the study. If the results show that you are not able to continue participating, the study doctor(s) will let you know:

- **EEG:** electroencephalography: this test allows us to look at your brain activity in real time
- **TMS:** transcranial magnetic stimulation: this procedure involves sending magnetic pulses to your brain to measure brain activity while we record with EEG (TMS-EEG)
- **TBS:** theta-burst stimulation: a newer version of rTMS that shortens the time of your daily treatment sessions
- **MRI:** magnetic resonance imaging: this procedure involves taking images of your brain in a scanner that will be then used to target the TBS treatment

*Questionnaires*

You will be provided with questionnaires to complete before you begin the study, which you will repeat at the end of the study, as well as at follow-up appointments. Some questionnaires will also consist of a short interview with a member of the research team. You will also complete a short checklist after every treatment session. The purpose of the questionnaires is to understand how TBS and illness affect your quality of life. The daily checklist should take a few minutes to complete, whereas the series of questionnaires that you will be completing prior to the study (as well as after the study) can take anywhere between 30-60 minutes to complete.

The information you provide is for research purposes only. Some of the questions are personal. You can choose not to answer questions if you wish.

Even though you may have provided information on a questionnaire, the responses will not be reviewed by your health care team or study team - if you wish for them to know this information please bring it to their attention.

*Detailed Procedures*

*Telephone Pre-Screening*

You already participated in a short phone-screening to determine your suitability, where we asked about your demographic information and screened you for any exclusion criteria. Verbal consent was obtained from you during that phone call

*Physician consultation*

- You have already met with one of the study physicians who has responded to your questions about the trial and has determined you are suitable for rTMS based on a short clinical assessment.

*Screening*

The study will include two visits prior to starting the TBS treatment trial. These sessions will be conducted closely together, approximately 1 week before the start of the treatment.

*Clinical Assessment*

- You will take part in a clinical evaluation to measure your overall severity and improvement of symptoms including depressive symptoms, anxiety symptoms, suicidal ideation, manic symptoms and quality of life.
- For some measures, you will answer questions on a tablet, and for others, we will verbally ask you some questions.
- We will also ask questions about your past and current medical history. If available, we may complement with information from your medical record, such as diagnosis, medication and date of entry.
- This will last approximately 90 minutes.

*MRI scan*

- You will undergo of 40 minutes scan at the Brain Imaging Centre.
- You will be asked to lay in a scanner while we take pictures of your brain.
- Prior to the scan session, you will be given the opportunity to practice for the scanning session using the Brain Imaging Center mock scanner so you can familiarize yourself with the scanner environment.

*rTMS intensity and measurements*

- You will be seated comfortably in a TMS therapy chair where the intensity used for all your treatment sessions will be determined by stimulating the brain with electrodes on your head.This will last approximately 15 minutes.
- Following this, We will take measurements using electromyographic (EMG). This will last approximately 10 minutes.

*Urine sample*

- A urine sample will be collected to screen for drug consumption and for pregnancy (female only)
- This will last approximately 10 minutes.

Phase 1: TBS Treatment Trial

*TBS Treatment Sessions*

- You will come to the clinic 5 days a week over the course of 4-6 weeks, for a total of 20-30 sessions. Sessions will run on the weekdays only, excluding stat holidays.
- You will be sitting comfortably on a reclining chair.
- The stimulating coil will be positioned over your forehead and TBS will be applied for 4 minutes.
- A short side effect questionnaire will be provided to you on a tablet to complete after the treatment session.
- The total duration of the treatment session will be approximately 15 minutes.

*Weekly Clinical Assessments*

- After every 5^th^ TBS session, you will be asked to fill questionnaires on a tablet to assess your clinical symptoms.
- Following this, a research team member will conduct a short interview to assess your symptoms.
- The total duration of these clinical assessments will be approximately 30 minutes.
- You will have one clinical assessment with a research team member to assess your response to the treatment during week 2 (half-way) which will last for about 30 minutes

*End of Treatment Clinical Assessments*

- After 20 sessions (at 4 weeks), you will undergo a full clinical assessment that will be similar to your baseline clinical assessment.
- It will consist of a series of questionnaires to complete on a tablet, a short interview with a research team member, followed by a consultation with the study psychiatrist if you are in remission. The total duration will be 1 hour.
  - If you are in remission, you will not receive further treatment sessions, and you will move on to the maintenance phase of the trial.
  - If you are not in remission, treatment will continue for another two weeks until 30 treatment sessions have been completed (after 6 weeks).
- After 30 sessions (at 6 weeks), you will undergo a full clinical assessment identical to the one received at week 4, followed by a consultation with the study psychiatrist.
  - If the clinical assessment shows that you benefited from treatment – you will move on to the Maintenance Phase.
- If the clinical assessment shows that you did not benefit from treatment – you will be discharged to your referring physician with a summary of treatment responses and recommendations.

*Measures of Brain Activity*

- During the first TBS session, at week 4 and week 6, we will measure your brain activity before and after you receive TBS.
- The stimulating coil will be positioned over the region of your brain that controls movement of your right index finger (about 7.5 centimeters above your left ear).
- A few stimulations will be delivered to determine the intensity of stimulation that will be used for TBS. You might feel a slight twitch in the muscle of your right index finger during stimulation. This is safe and painless
- Then, a cap containing 64 small flat electrodes will be positioned over your head. A very small amount of gel will be inserted into each electrode, touching your scalp.
- We will position the coil over your left and right forehead regions, and deliver magnetic pulses while we will record the activity of your brain using a technique called electroencephalography (EEG).
- TBS will then be applied through the coil.
- Immediately after, we will again stimulate your left and right forehead.
- These three visits will last approximately 120 minutes.

Phase 2: Maintenance Treatment Trial

*Frequency of TBS sessions*

- After completing the 20-30 TBS treatment sessions, you will undergo a 6-month maintenance phase.
- The TBS sessions will be identical to the Treatment Phase. A short clinical assessment will be conducted at a regular frequency. The session duration will vary from 15 to 35 minutes.
- If you are assigned a **flexible** maintenance protocol:
  - Month 1: You will receive 2 sessions/week.
  - Months 2 and 3: we will conduct a short clinical assessment once a week in which we will determine if you will receive 0, 1 or 2 weekly TBS session.
  - Months 4 and 5: we will conduct a short clinical assessment twice in the month, in which we will determine if you will receive 0, 1 or 2 TBS session in the next two weeks.
  - Months 6: we will conduct a short clinical assessment at the beginning of the month in which we will determine if you will receive 0, 1 or 2 monthly TBS session.
  - You will also meet with the study psychiatrist on an as needed-basis to discuss your well-being and maintenance progress.

*Final TBS Session*

- At week 24 (end of month 6), all participants will have a final visit that will include:
  - A full clinical assessment (questionnaires, interview and physician consultation)
  - Measures of brain activity
- The total duration of this session will be 2 hours.
- You will be discharged to your referring physician with a summary of treatment response and recommendations.

WHAT ARE THE RESPONSIBILITIES OF STUDY PARTICIPANTS?

If you choose to participate in this study, you will be expected to:

- Tell the study doctor about your current medical conditions;
- Tell the study doctor if there are any changes in your treatment during the study (e.g. start of a new psychotherapy);
- Tell the study doctor about all prescription and non-prescription medications and supplements, including vitamins and herbals, and check with the study doctor before starting, stopping or changing any of these. This is for your safety as these may interact with the treatment you receive from this study;
- Tell the study doctor if you are thinking about participating in another research study;
- Tell the study doctor if you become pregnant or are breastfeeding while participating in this study.

HOW LONG WILL PARTICIPANTS BE IN THE STUDY?

The study intervention will last for 4-6 weeks, and the maintenance phase will last 6 months after your last treatment session, with total study commitment lasting 7 to 7.5 months. You may be seen more often if the study doctor determines that this is necessary.

CAN PARTICIPANTS CHOOSE TO LEAVE THE STUDY?

You can choose to end your participation in this research (called withdrawal) at any time without having to provide a reason. If you choose to withdraw from the study, you are encouraged to contact the study doctor or study staff.

You may be asked questions about your experience with the study intervention, and have laboratory tests and physical examinations considered necessary to safely stop your involvement.

You may withdraw your permission to use information that was collected about you for this study at any time by letting the study doctor know. However, this would also mean that you withdraw from the study.

If participants relapse (reach baseline assessment scores) in the maintenance phase, the researchers will have a discussion with the participant regarding their participation and schedule a consultation with a study physician to help determine if completion of the study is necessary at that time.

CAN PARTICIPATION IN THIS STUDY END EARLY?

The study doctor may stop your participation in the study early, and without your consent, for reasons such as:

- You are unable to tolerate the study treatment
- You are unable to complete all required study procedures
- New information shows that the study treatment is no longer in your best interest
- The study doctor no longer feels this is the best option for you (e.g. develop active suicidal ideation with intent or experience worsening symptoms)
- The Regulatory Authority/ies or Research Ethics Board withdraw permission for this study to continue
- If you plan to or become pregnant
- If you miss or fail to attend >3 consecutive treatment days or 15% of sessions during the treatment course
- If you experience a sustained relapse in the maintenance phase of the study, defined by requiring 2 treatments/week for more than 2 consecutive assessments
- If you have any psychotropic medication changes or changes to your current antidepressant regimen
- If you are lost to follow-up (i.e. lose contact)

If this happens, it may mean that you would not receive the study treatment for the full period described in this consent form.

If you are removed from this study, the study doctor will discuss the reasons with you and plans will be made for your continued care outside of the study.

WHAT ARE THE RISKS OR HARMS OF PARTICIPATING IN THIS STUDY?

You may experience side effects from participating in this study. The study doctor will watch you closely to see if you have side effects. Some side effects are known and are listed below, but there may be other side effects that are not expected. You should discuss these with the study doctor.

*1. Clinical Interview*

The topics discussed in the clinical interview may cause some emotional discomfort. We ask you to inform members of the research team if this is the case. The information collected from this interview and from questionnaires that you will complete, is treated professionally and confidentially. If the results of your questionnaires suggest the presence of concerning new symptoms, we will provide you with an appropriate referral.

*2. Risks Associated with TMS*

Tens of thousands of people have received rTMS treatments over the past 20 years. There are no known or predictable long-term risks associated with rTMS. rTMS is considered a very safe procedure, particularly since guidelines for its use were established in 1998. Its side effect profile compares to those of most antidepressant medication. Moreover, evidence indicates that rTMS is not associated with any negative effects on cognitive abilities such as memory or concentration. However, rTMS is associated with some known side effects that will be described below and divided in terms of how frequently they occur:

**Common:** mild discomfort or pain at the site of stimulation (40%), muscle-type tension headache (30%), facial muscle twitching (30%), lightheadedness or dizziness (20%). These side effects are usually temporary and can be effectively managed with rest, or over-the counter medication such as acetaminophen (Tylenol).

**Less common (1-7%):** fatigue, headache persisting after treatment, insomnia, anxiety or agitation, back or neck pain, vomiting, tinnitus (ringing in the ears), migraine aura or abnormal sensations. These side effects will be closely monitored after each rTMS session and the assigned clinician will be contacted if any of these arise.

**Rare but serious (<1%):** onset of suicidal thinking, worsening of depressive symptoms or treatment-emergent mania (opposite of depression with symptoms of elevated mood, increased energy, impulsiveness and decreased need for sleep). You will be monitored regularly for these symptoms during treatment and will have immediate access to the assigned study psychiatrist if they appear.

**Very rare but serious (<0.1%):** There are rare cases of an epileptic seizure resulting from rTMS. Most of these (about 16 episodes since 1985) occurred before 1998, when settings that we now know to be unsafe, were used. In 1998, safety guidelines for TMS were adopted, and since then, only 5 additional episodes of seizure have occurred in the world. In most cases, patients were taking medications known to modify brain activity. All seizures that occurred stopped spontaneously with no long-term effects. Importantly, no one has ever developed epilepsy after an rTMS induced seizure. Seizure monitoring procedures will be implemented during this study (e.g., visual monitoring for signs of seizures). In the unlikely case that a participant experiences a seizure, the study will be terminated and our staff will immediately inform 911 and have an ambulance take you to the emergency services.

Induced syncope (fainting) may also arise in the first rTMS sessions, related to anxiety or discomfort and therefore not directly caused by rTMS. Physical manifestations of syncope can mimic symptoms of seizures, such as brief head or eye version. As such, research staff will be trained to respond to syncope using the same procedure as for a suspected epileptic seizure.

*3. Risks associated with TMS-EEG:*

Side effects of single pulse TMS are similar to rTMS, but risk of seizure is even lower. Regarding EEG, the risks involved are minimal. EEG monitoring procedures conducted in this study are similar to those carried out in hospitals. Slight redness may occur where electrodes are placed on the skin and scalp. Participants may also experience boredom or restlessness during recordings as there are no tasks involved.

*4. Risks associated with the MRI Scan*

Some participants may experience discomfort while trying to remain motionless inside the scanner. Feelings of claustrophobia (i.e., the fear of enclosed spaces) may arise while inside the scanner or by the noise levels made by the scanner during the study. You will be provided with both earplugs and earphones to help reduce scanner noise exposure. Any discomfort experienced is generally mild. The technologist and research team staff will be able to see and hear you throughout the scan. You will be given a squeeze ball that you can squeeze at any time during the scan to let the staff know that you would like to be removed from the scanner.

WHAT ARE THE REPRODUCTIVE RISKS?

There are no known risks of rTMS during pregnancy. However, there is always a possibility that if you are pregnant, rTMS may have risks that we do not know about. For this reason, you should not participate in the study if you may be pregnant. Pregnancy will be assessed with a urine test as part of your screening. Women should also not nurse (breastfeed) a baby for the total duration of the study.

WHAT ARE THE BENEFITS OF PARTICIPATING IN THIS STUDY?

If you agree to take part in this study, the experimental intervention may or may not be of direct benefit to you. We approximate that one in two participants will benefit from TBS. By participating in this study, you will have free access to an evidence-based treatment for major depression that is not currently offered in the Ottawa/Gatineau region. In addition, you will help us determine which individuals may be more likely to respond to unilateral versus bilateral treatment. This project will also help us explore predictors and outcome measures of treatment response, essential in the development of better treatment options.

HOW WILL PARTICIPANT INFORMATION BE KEPT CONFIDENTIAL?

If you decide to participate in this study, the study doctors and study staff will only collect the information they need for this study.

Records identifying you at this centre will be kept confidential and, to the extent permitted by the applicable laws, will not be disclosed or made publicly available, except as described in this consent document.

Authorized representatives of the following organizations may look at your original (identifiable) medical/clinical study records at the site where these records are held, to check that the information collected for the study is correct and follows proper laws and guidelines.

- Members of the research team will need to access your records to determine if you meet the requirements to participate in this study.
- Representatives from the Royal’s Institute of Mental Health Research who oversee the safety and quality of this study.
- The Royal’s Research Ethics Board, to oversee the ethical conduct of research at this location

Information that is collected about you for the study (called study data) may also be sent to the organizations listed above. Your name, address, or other information that may directly identify you will not be used. The records received by these organizations may contain your participant code, initials, sex, and date of birth.

Studies involving humans sometimes collect information on race and ethnicity as well as other characteristics of individuals because these characteristics may influence how people respond to different interventions. Providing information on your race or ethnic origin is voluntary.

This study requires the transfer of identifiable information to your referring for the purposes of summarizing your response to treatment. The following information will be transferred:

- A fax containing your name and a summary of response to treatment and treatment recommendations at the end of your participation in the study

If the results of this study are published, your identity will remain confidential. It is expected that the information collected during this study will be used in analyses and will be published/ presented to the scientific community at meetings and in journals. This information may also be used as part of a submission to regulatory authorities around the world to support the approval of the study intervention.

Even though the likelihood that someone may identify you from the study data is very small, it can never be completely eliminated.

A copy of the consent form that you sign to enter the study may be included in your health record/hospital chart.

WILL FAMILY DOCTORS/HEALTH CARE PROVIDERS KNOW WHO IS PARTICIPATING IN THIS STUDY?

Your family doctor/health care provider referred you to this study and will be informed that you are taking part in a study so that you can be provided with appropriate medical care. If you do not want your family doctor/health care provider to be informed of the outcome of your treatment, please discuss this with the study team.

WILL information about this study BE available online?

A description of this clinical trial will be available on <http://www.clinicaltrials.gov> (registration number: NCT04142996). This website will not include information that can identify you. You can search this website at any time.

WHAT IS THE COST TO PARTICIPANTS?

TBS will be administered at no charge while you take part in this study.

You may not be able to receive the study intervention after your participation in the study is completed. There are several possible reasons for this, some of which are:

- The intervention, even if approved in Canada, may not be available free of charge.

The study doctor will talk to you about your options.

Taking part in this study may result in added costs to you. For example:

- There may be costs associated with hospital visits. For example, parking or transportation, or snacks/meals during your stay.
- You may miss work as a result of participation in this study.

ARE STUDY PARTICIPANTS PAID TO BE IN THIS STUDY?

You will be compensated for your time during the full clinical assessments and the TMS-EEG session. However, you will not receive compensation for the TBS treatment sessions. During the treatment trial, you will receive $30 for the baseline MRI scan, and $20 compensation for each of the clinical assessments (at the baseline visit, the end of week 4, and the end of week 6 if applicable). You will also be compensated $20 for each of the TMS-EEG measurements (the first session and either the end of week 4 OR the end of week 6 depending on your response level to the treatment). Therefore, you will receive a total of $100 to $120 (depending on if you receive treatment for 4 or 6 weeks).

In the Maintenance Phase, you will receive compensation of $5 for the short weekly or monthly clinical assessments, as well as $20 for the final clinical assessment and $20 for the final TMS-EEG measurement. As such, you will receive a total of $125 for your participation in the maintenance phase of the study.

If needed or requested by participant, a parking coupon can be provided for the study visit or alternatively, public transit costs within Ottawa can be covered (upon presentation of the receipt) for the visits in which they do not receive monetary compensation. Note that there is free parking available in adjacent streets and that we will not reimburse public transit in the case where participants have a monthly pass.

In the case of research-related side effects or injury, medical care will be provided by the study doctor or you will be referred for appropriate medical care.

WHAT ARE THE RIGHTS OF PARTICIPANTS IN A RESEARCH STUDY?

You will be told, in a timely manner, about new information that may be relevant to your willingness to stay in this study.

You have the right to be informed of the results of this study once the entire study is complete. If you would like to be informed of the results of this study, please contact the study doctor. In addition, the results of this study will be available on the clinical trial registry (see the “Will information about this study be available online” section for more details).

Your rights to privacy are legally protected by federal and provincial laws that require safeguards to ensure that your privacy is respected.

By signing this form you do not give up any of your legal rights against the study doctor, or involved institutions for compensation, nor does this form relieve the study doctor, or their agents of their legal and professional responsibilities.

You will be given a copy of this signed and dated consent form prior to participating in this study.

WHAT IF RESEARCHERS DISCOVER SOMETHING ABOUT A RESEARCH PARTICIPANT?

During the study, the researchers may learn something about you that they didn’t expect. For example, the researchers may discover that you have another medical condition.

If any new clinically important information about your health is obtained as a result of your participation in this study, you will be given the opportunity to decide whether you wish to be made aware of that information.

WHOM DO PARTICIPANTS CONTACT FOR QUESTIONS?

If you have questions about taking part in this study, or if you suffer a research-related injury, you can talk to your study doctor, or the doctor who is in charge of the study at this institution. That person is:

Sara Tremblay, PhD 613-722-6521 ext. 6227

____________________________ _________________________

Name Telephone

This study has been reviewed and approved by the Royal’s Institute of Mental Health Research REB as study #2019017. If you have any ethical concerns about the study, or the way it is conducted, please contact the REB office: [Tammy.Beaudoin@theroyal.ca](mailto:Heidi.Vulin@theroyal.ca)

SIGNATURES

- All of my questions have been answered,
- I understand the information within this informed consent form,
- I allow access to my medical records as explained in this consent form,
- I do not give up any of my legal rights by signing this consent form,
- I understand that my family doctor/health care provider will be informed of my participation in this study,
- I agree, or agree to allow the person I am responsible for, to take part in this study.

Anonymized data (identifiers removed) will be stored on the IMHR secure network and password protected. This anonymized data collected in the study can be shared with other IMHR Researchers for combining with existing data to better understand the pathophysiology of mental health disorders. The Principal Investigator (Dr. Sara Tremblay) will be responsible for the data and its distribution. Do you agree to share your anonymized data for future research purposes: Yes ☐ No ☐

__________________________ ______________________ ______________

Signature of Participant PRINTED NAME Date

____________________________ ______________________ ______________

Signature of Person Conducting PRINTED NAME & ROLE Date

The Consent Discussion

**Complete the following section only if the participant is unable to read or requires an oral translation:**

- The informed consent form was accurately explained to, and apparently understood by, the participant, and
- Informed consent was freely given by the participant

____________________________ ______________________ ______________

Signature of Impartial PRINTED NAME Date

Witness/Translator

*(If participant were unable to*

*read/required an oral translation)*

**PERMISSION TO BE CONTACTED FOR RESEARCH**

The University of Ottawa's Institute of Mental Health Research (IMHR) is committed to building a future where we can identify and successfully treat mental illness.

We are asking you for your permission to allow approved research staff to contact you to see if you are interested in participating in a research study being conducted at the IMHR.

Even if you provide your permission to be contacted now, you may withdraw your permission at any time.  If you prefer not to be contacted for research, your care and treatment will not be affected in any way.

Any personal health information you may give us is protected under the Personal Health Information Protection Act, 2004 (Ontario).

**SIGNATURE FOR FUTURE STUDY CONTACT**

- I understand that studies may want to contact me in the future to see if I am interested in additional study components or other studies I may be suitable for and that I need to provide my permission that approved research staff may contact me. I know that I can change my mind by notifying current study staff and that I can decline future study participation.
- I will be asked if I want a copy of this permission to contact form to bring home.

**I FREELY ACCEPT TO BE CONTACTED IN THE FUTURE ABOUT OTHER STUDY OPPORTUNITIES**

__________________________ ______________________ ______________

Signature of Participant PRINTED NAME Date

____________________________ ______________________ ______________

Signature of Person Conducting PRINTED NAME & ROLE Date

the Consent Discussion
